# Gene-environment interaction explains a part of missing heritability in human body mass index

**DOI:** 10.1038/s42003-023-04679-4

**Published:** 2023-03-25

**Authors:** Hae-Un Jung, Dong Jun Kim, Eun Ju Baek, Ju Yeon Chung, Tae Woong Ha, Han-Kyul. Kim, Ji-One Kang, Ji Eun Lim, Bermseok Oh

**Affiliations:** 1 Department of Biomedical Science, Graduate School, Kyung Hee University, Seoul, South Korea; 2 Department of Biochemistry and Molecular Biology, School of Medicine, Kyung Hee University, Seoul, South Korea

**Keywords:** Diseases, Medical research

## Abstract

Gene-environment (G×E) interaction could partially explain missing heritability in traits; however, the magnitudes of G×E interaction effects remain unclear. Here, we estimate the heritability of G×E interaction for body mass index (BMI) by subjecting genome-wide interaction study data of 331,282 participants in the UK Biobank to linkage disequilibrium score regression (LDSC) and linkage disequilibrium adjusted kinships–software for estimating SNP heritability from summary statistics (LDAK-SumHer) analyses. Among 14 obesity-related lifestyle factors, MET score, pack years of smoking, and alcohol intake frequency significantly interact with genetic factors in both analyses, accounting for the partial variance of BMI. The G×E interaction heritability (%) and standard error of these factors by LDSC and LDAK-SumHer are as follows: MET score, 0.45% (0.12) and 0.65% (0.24); pack years of smoking, 0.52% (0.13) and 0.93% (0.26); and alcohol intake frequency, 0.32% (0.10) and 0.80% (0.17), respectively. Moreover, these three factors are partially validated for their interactions with genetic factors in other obesity-related traits, including waist circumference, hip circumference, waist-to-hip ratio adjusted with BMI, and body fat percentage. Our results suggest that G×E interaction may partly explain the missing heritability in BMI, and two G×E interaction loci identified could help in understanding the genetic architecture of obesity.

## Abstract (web summary)

A study on the effects of gene-environment interactions on body mass index (BMI) in the UK Biobank cohort finds BMI genetic loci that interact with lifestyle traits such as physical activity, smoking, and alcohol consumption.

## Introduction

Genome-wide association studies (GWASs) have uncovered several genetic variants that affect complex traits^1–3^, enhanced our understanding of the development of diseases, and provided genetic targets for the treatment of diseases^2,4^. However, the effect sizes of individual variants discovered by GWAS are relatively small—in the range of 1.01–1.20—such that the polygenic risk score (PRS), summing up all effects of GWAS single nucleotide polymorphisms (SNPs), explains less than 10% of the phenotypic variance in most traits^4–7^. Therefore, heritability (the estimated genetic effects on traits) based on SNPs was far lower than that estimated by traditional analyses based on twin and family studies^7^, even though they may be inflated due to confounding with shared environmental effects with in families or twins^8^. Recent studies, including those using the Genome-wide complex trait analysis, a method calculating the SNP heritability from all possible common variants, suggested that common genetic variants can explain up to 70% of the variation in height and 40% of the variation in body mass index (BMI)^9–12^. Despite these progresses, substantial missing heritability remains in most complex traits^7,13–16^.

Complex traits are also affected by environmental factors, and the environmental effect can modify genetic risk factors, known as gene-environment (G × E) interaction^17–21^. Genetic variants within the *FTO* gene that exhibit the largest effect size on obesity were found to interact with environmental factors, such as physical activity, diet, alcohol consumption, and sleep duration^22–27^. The effect size of *FTO* variants on BMI was reduced by increased physical activity and enhanced by decreased physical activity^22–25^. These findings indicate that genetic susceptibility to disease can be modulated by altering environmental factors^22,25,28,29^, and also suggest that the G × E interaction may contribute to missing heritability^30^.

Generally, it is difficult to accurately measure environmental exposures, because many cases depend on self-reported questionnaires for past exposures^29,31^. In addition, the analysis of G × E interaction generally requires a higher sample size than that used in genetic association analysis^20^. Therefore, the identification of genetic variants having true G × E interaction effects in complex human traits has been challenging^21,31–33^. Nevertheless, recent progress in the analysis of G × E interaction has shed light on the importance of their effect on trait phenotype. Poveda et al. investigated the G × E interaction in cardiometabolic traits in the VIKING study, which involved a cohort of 16,430 Swedish adults from 1682 extended pedigrees^34^. They found statistically significant effects of gene-age and gene-alcohol intake interactions on weight, as well as effects of gene-age interactions on systolic blood pressure, using quantitative genetic analysis in extended pedigrees. Further, Justice et al. performed a genome-wide analysis of gene-smoking interaction on obesity-related traits in 241,258 samples (51,080 current smokers and 190,178 nonsmokers) to understand the effects of smoking on genetic susceptibility to obesity^35^. They identified 31 genetic loci for gene-smoking interaction, and gene set analysis using these genetic loci revealed various pathways, including response to oxidative stress and addictive behavior, where dysregulation may lead to increased susceptibility to obesity. In addition, two studies investigated the presence of G × E interaction in BMI and estimated genetic risk using PRS calculated from GWAS statistics^28,29^. They used the UK Biobank GWAS data, which include 500,000 participants with lifestyle measurements, and found significant G × E interactions of PRS with physical activity, alcohol consumption, and socioeconomic status in BMI. These studies demonstrate the statistical significance of the G × E interaction in complex traits. However, it is still unclear how much the G × E interaction can explain the effect of phenotypic variance on the extent of heritability.

Recently, two studies estimated the extent by which the G × E interaction explained the phenotypic variance of obesity. Robinson et al. estimated the difference in the heritability of G × E interaction in eight self-reported lifestyle variables, including diet, exercise, and smoking^36^. They found an evidence for the genetic interaction effect with smoking behavior, which was estimated to contribute 4.0% to the BMI variation. Sulc et al. investigated the contribution of G × E interaction to obesity-related traits from the UK Biobank using a maximum likelihood method^37^. Previously, Wang et al. showed the presence of G × E interaction by estimating the difference in phenotype variance in different genotype groups of subjects^31^. Sulc et al. applied the assumption of Wang et al. that the estimation of G × E interaction in a genome-wide level is presumably affected by all environmental risk factors. Using this approach, Sulc et al. found that G × E interaction effects of genome-wide SNPs explained 1.9% of the variance in BMI in addition to the 15% contributed by genetic risk factors. However, Sulc et al. did not use the real measurements of environmental factors; therefore, their findings for the effect size of G × E interaction on BMI require validation.

In this study, we aimed to (1) provide evidence for the heritability of G × E interaction for BMI using real environment measurements, (2) estimate the effect size of the G × E interaction heritability for BMI, and (3) identify novel genetic loci that interact with environmental factors to affect BMI. We used the UK Biobank data for this analysis, which included 331,282 participants with 4,143,506 SNPs and 14 obesogenic lifestyles. To calculate G × E interaction heritability, we used both linkage disequilibrium score regression (LDSC) and linkage disequilibrium adjusted kinships–software for estimating SNP heritability from summary statistics (LDAK-SumHer)^38,39^.

## Results

### Basic characteristics of participants related to BMI

We selected unrelated 331,282 UK Biobank participants of “White-British” European ancestry for this study, similar to those used in the Neale lab (https://github.com/Nealelab/UK_Biobank_GWAS). The lifestyle characteristics of the 331,282 participants are summarized in Table 1. The participants were divided into quartiles based on their BMI values: men and women were separately divided into quartiles and then the men and women in the same quartile group were combined into one responding quartile group. The average BMI of all participants was 27.39 (SD = 4.75), and the average BMIs were 22.32 (SD = 1.62) for the first, 25.40 (SD = 1.01) for the second, 28.10 (SD = 1.02) for the third, and 33.75 (SD = 3.85) for the fourth quartile groups.

**Table 1:** Basic characteristics of UK Biobank participants included in this study.

Group	Quartile 1 group	Quartile 2 group	Quartile 3 group	Quartile 4 group
Female threshold	BMI ≤ 23.42	23.42 < BMI ≤ 26.06	26.06 < BMI ≤ 29.63	29.63 < BMI
Male threshold	BMI ≤ 24.99	24.99 < BMI ≤ 27.30	27.30 < BMI ≤ 30.04	30.04 < BMI
Number of participants	82,869	82,799	82,823	82,791
Males (%)	46.23	46.26	46.21	46.24
Age at assessment center (years) (mean, SD)	55.81 ± 8.18	56.89 ± 7.98	57.40 ± 7.86	57.16 ± 7.72
Body mass index (kg/m^2^) (mean, SD)	22.32 ± 1.62	25.40 ± 1.01	28.10 ± 1.02	33.75 ± 3.85
Met score				
Met score mean (SD)	2895.06 ± 2771.15	2768.21 ±2699.87	2657.59 ± 2719.93	2323.56 ±2603.84
Moderate Physical activity				
Frequency mean (SD)	3.87 ± 2.31	3.72 ± 2.29	3.58 ± 2.31	3.29 ± 2.36
Time spent watching television (TV)				
Spent time mean (SD)	2.35 ± 1.55	2.66 ± 1.52	2.92 ± 1.58	3.29 ± 1.75
Time spent using computer				
Spent time mean (SD)	0.98 ± 1.24	1.02 ± 1.26	1.06 ± 1.33	1.16 ± 1.47
Smoking status (%)				
Never	59.35	56.11	52.72	50.76
Previous smoker	29.03	34.27	37.69	39.95
Current smoker	11.62	9.61	9.6	9.28
Pack years of smoking				
Pack years of smoking mean (SD)	6.26 ± 13.49	7.09 ± 14.11	8.83 ± 15.84	11.12 ± 18.87
Alcohol intake status (%)				
Never	2.93	2.48	2.88	3.93
Previous	3.25	2.69	3.19	4.46
Current	93.82	94.83	93.93	91.61
Alcohol intake frequency				
Category mean (SD)	2.70 ± 1.47	2.69 ± 1.42	2.82 ± 1.46	3.16 ± 1.52
Neuroticism score				
Neuroticism score mean (SD)	4.17 ± 3.26	3.99 ± 3.20	4.06 ± 3.25	4.23 ± 3.30
Fed-up feelings				
Yes (%)	36.69	37.35	40.88	47.42
Sleep duration				
Sleep duration mean (SD)	7.18 ± 1.01	7.19 ± 1.02	7.18 ± 1.09	7.13 ± 1.21
Nap during day (%)				
Never/rarely	63.1	59.13	55.33	49.07
Sometimes	32.78	36.4	39.5	43.96
Usually	4.12	4.47	5.17	6.97
Average total household income before tax				
Category mean (SD)	2.76 ± 1.20	2.71 ± 1.18	2.60 ± 1.18	2.44 ± 1.16
Townsend deprivation index (TDI)				
Townsend deprivation index mean (SD)	−1.68 ± 2.91	−1.83 ± 2.79	−1.67 ± 2.85	−1.14 ± 3.10

Data, mean ± standard deviation (SD).

*BMI* body mass index, *MET score* metabolic equivalent of task score.

Physical activity (category ID: 100054), metabolic equivalent task (MET) scores (category ID: 54), mental health (category ID: 100060), smoking (category ID: 100058), alcohol (category ID: 100051), sleep (category ID: 100057), household (category ID: 100066), and baseline characteristics (category ID: 100094) have been repeatedly reported to be related to obesity^24,29,40^. We selected 14 lifestyle factors among these categories for this study, based on previous reports, while ensuring a sample size of at least 250,000 individuals with lifestyle measures^29^. The 14 lifestyle variables are shown in Table 1, and the field IDs of these lifestyle variables are described in the Materials and Methods. The average values of lifestyle variables or the percentages in each BMI group are also provided in Table 1. Moreover, the distributions of the 14 lifestyle variables in all 331,282 participants are depicted as histograms, and both distributions of raw and processed variables for normalization are shown in Supplementary Fig. 1. The method for the processing of raw data is described in the Materials and Methods.

We performed association analysis between BMI as a continuous trait and the 14 lifestyle factors using linear regression (Supplementary Table 1). All 14 lifestyle factors showed a significant association under multiple testing (*P* < 3.57 × 10^−3^) with BMI adjusted for age and sex in both raw and processed variables. We found positive correlations between BMI and pack years of smoking, smoking status, time spent watching TV, fed-up feelings, neuroticism score, townsend deprivation index at recruitment (TDI), nap during day, alcohol intake frequency, and time spent using computer. In contrast, negative correlations were seen between BMI and MET score, sleep duration, moderate physical activity, alcohol intake status, and average total household income before tax (Supplementary Table 1). The analysis of lifestyle factors in the physical activity and MET category, such as summed MET minutes per week for all activity, moderate physical activity, time spent using computer, and time spent watching television, clearly demonstrated that participants with more active lifestyles tended to have a lower BMI, and participants with sedentary lifestyles exhibited a higher BMI. However, the relationships of other lifestyle factors with BMI were not as clear (Table 1). Moreover, we assessed the correlation coefficients between BMI and lifestyle factors as well as between lifestyle factors using a correlogram (Supplementary Fig. 2).

### Effect of G × E interaction on BMI

**Fig. 1:**
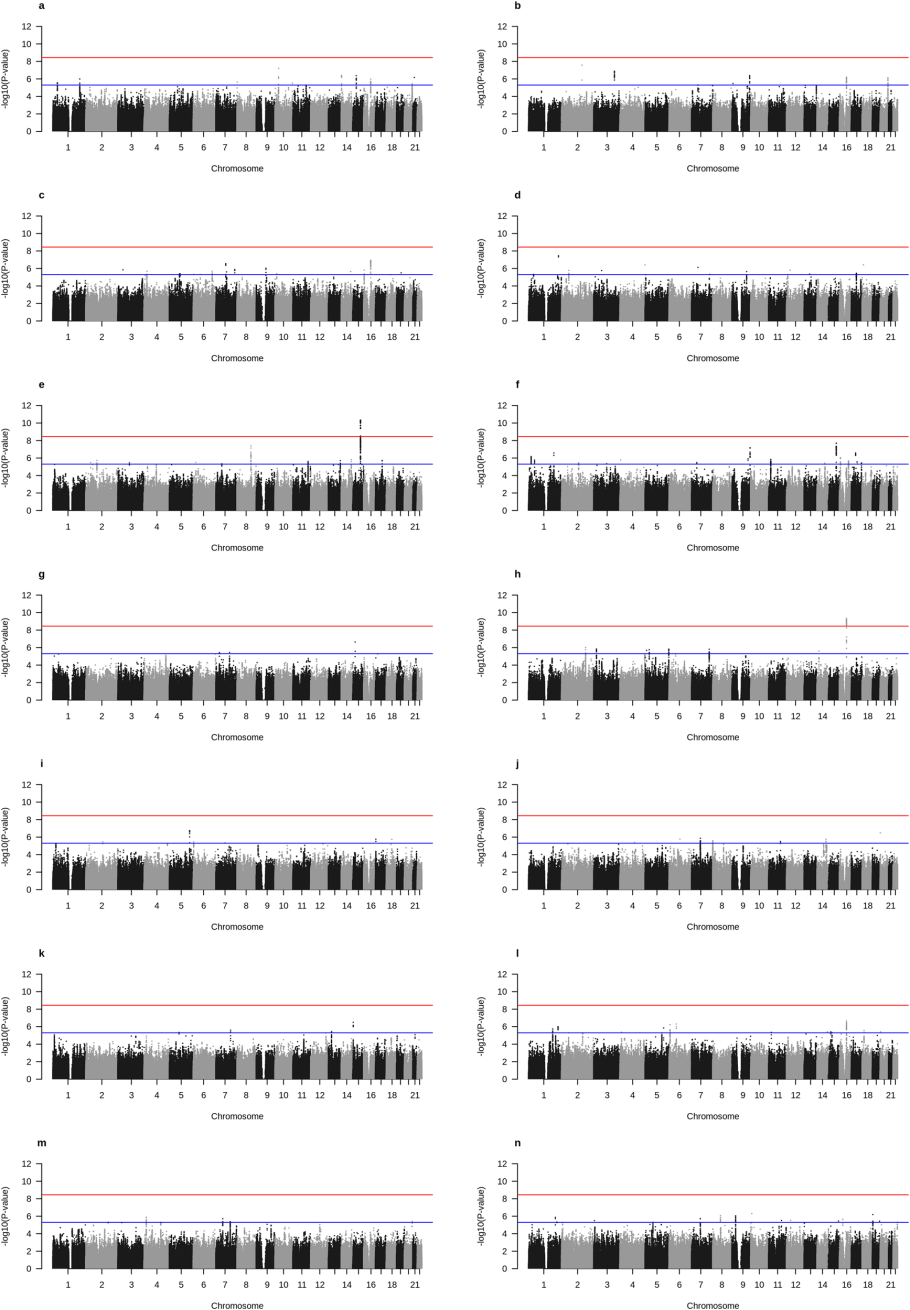
Manhattan plots of gene-environment interaction study. Manhattan plots showing the –log_10_-transformed gene-environment interaction *P*-value of each SNP on the *y*-axis and base-pair positions along the chromosomes on the *x*-axis. SNP *P*-values were computed in Plink 1.90. The blue line indicates the suggestive threshold (*P* < 5.00 × 10^−6^). The red line indicates the genome-wide significance for multiple testing (*P* < 3.57 × 10^−9^). **a** Metabolic equivalent task (MET) score, **b** Moderate physical activity, **c** Time spent wathcing television (TV), **d** Time spent using computer, **e** Smoking status, **f** Pack years of smoking, **g** Alcohol intake status (the GWIS after genomic control), **h** Alcohol intake frequency (the GWIS after genomic control), **i** Neuroticism score, **j** Fed-up feelings, **k** Sleep duration (the GWIS after genomic control), **l** Nap during day, **m** Average total household income before tax, **n** Townsen deprivation index at recruitment.

We tested the effect of G × E interactions between the 4,143,506 SNPs (minor allele frequency [MAF] ≥ 0.05) and individual lifestyle factors on BMI as a continuous trait, using fixed effect models of linear regression (PLINK v.1.90). To investigate genomic inflation, we estimated the genomic control lambda and the intercept value of LD score regression on each GWIS result (Supplementary Table 2). The intercept value of LD score regression indicates statistical inflation adjusted for LD structure and is considered as a tool for a more powerful and accurate correction than the genomic control lambda^38^. Since the intercept value of alcohol intake status (1.38), alcohol intake frequency (1.17), and sleep duration (1.17) were higher than 1.1, we applied the genomic control correction using lambda values to these lifestyle factors. As a result, the intercept values become decreased after the correction: alcohol intake status, 0.99; alcohol intake frequency; 0.96; sleep duration 0.98 (Supplementary Table 2). The results of G × E interaction using processed variables are depicted in Fig. 1 as Manhattan plots and in Supplementary Fig. 3 as quantile–quantile (Q–Q) plots. We found two genome-wide significant signals for multiple testing (*P* < 3.57 × 10^−9^ calculated by 5.00 × 10^−8^/14) in the lifestyle factors analyzed, as shown in Supplementary Table 3. The suggestive associations of G × E interaction with *P* < 5.00 × 10^−6^ are provided in Supplementary Table 4.

Further, we investigated functional annotation of these two G × E interaction lead SNPs. First, we searched for functional importance of these SNPs using RegulomeDB (Supplementary Table 5). Second, we evaluated any association of genetic variants existing within the 1 Mb flanking region of the lead SNPs with obesity or obesity-related traits (Supplementary Table 6). The direct association of lead SNPs with any traits was also determined using the GWAS catalog (Supplementary Table 7). Third, we investigated the expression quantitative trait loci (eQTL) information using Genotype-Tissue Expression (GTEx) version 8 for lead SNPs shown genome-wide significance level (Supplementary Table 8)^41^. All two lead SNPs had genetic variants within the 1 Mb flanking region associated with BMI. The direct association of rs11642015 (interacting with alcohol intake frequency) with the G × E interaction in BMI and that of rs12438181 (interacting with smoking status) with pulmonary function in smokers were reported, and two lead SNPs showed eQTL genes as sentinel SNPs (*r*^2^ = 1).

### Heritability of G × E interaction

Based on the results of genome-wide G × E interaction tests, we calculated the heritability of G × E interaction for BMI using LDSC v1.0.1 (https://github.com/bulik/ldsc) (Table 2)^38^. Among the 14 lifestyle factors, MET score (*P* = 3.38 × 10^−3^), time spent watching television (*P* = 1.13 × 10^−3^), pack years of smoking (*P* = 1.74 × 10^−4^), alcohol intake frequency (*P* = 1.26 × 10^−6^), and fed-up feeling (*P* = 2.24 × 10^−3^) showed statistical significance, based on the Bonferroni multiple correction (*P* < 3.57 × 10^−3^) (Table 2). The heritability of G × E interaction for alcohol intake frequency in BMI was *h*_G×E_^2^ = 0.0080, which suggests that 0.80% of the BMI phenotypic variance in the population might be attributed to the genetic interaction with alcohol intake frequency. Similarly, the heritability of G × E interaction for MET score was *h*_G×E_^2^ = 0.0065, that of time spent watching TV was *h*_G×E_^2^ = 0.0058, that of pack years of smoking was *h*_G×E_^2^ = 0.0093, and that of fed-up feelings was *h*_G×E_^2^ = 0.0054.

**Table 2:** G × E interaction heritability in BMI calculated using the LDSC method.

Category	Lifestyle factor	*N*	G × E interaction heritability % (SE)	*P*-value	Intercept
Physical activity	MET score^a^	268,536	0.65 (0.24)	3.38 × 10^−3^	1.084
	Moderate physical activity	315,747	0.41 (0.16)	5.20 × 10^−3^	1.025
Physical activity	Time spent watching television (TV)^a^	328,943	0.58 (0.19)	1.13 × 10^−3^	1.090
(Leisure life)	Time spent using computer	328,824	1.22 × 10^−3^ (0.21)	5.00 × 10^−1^	1.087
Smoking	Smoking Status	330,138	0.31 (0.18)	4.17 × 10^−2^	1.086
	Pack years of smoking^a^	279,758	0.93 (0.26)	1.74 × 10^−4^	1.070
Alcohol	Alcohol intake status^b^	330,995	0.32 (0.17)	2.99 × 10^−2^	0.985
	Alcohol intake frequency^a,b^	331,049	0.80 (0.17)	1.26 × 10^−6^	0.960
Mental health	Neuroticism score	269,392	0.52 (0.22)	9.05 × 10^−3^	1.040
	Fed-up feelings^a^	324,753	0.54 (0.19)	2.24 × 10^−3^	1.023
Sleep	Sleep duration^b^	329,523	0.09 (0.16)	2.87 × 10^−1^	0.981
	Nap during day	331,141	0.18 (0.19)	1.70 × 10^−1^	1.060
Social economic status	Average total household income before tax	285,544	0.28 (0.18)	5.99 × 10^−2^	0.996
	Townsend deprivation index at recruitment	330,893	0.42 (0.17)	6.70 × 10^−3^	1.083

*MET score* metabolic equivalent of task score, *SE* standard error.

^a^Indicates the statistically significant G × E interaction heritability based on the Bonferroni corrected *P*-value threshold (*P*-value < 3.57 × 10^−3^).

^b^Indicates the GWIS after genomic control.

**Fig. 2:**
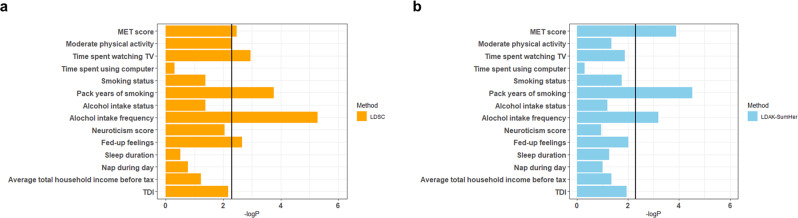
Bar plots of G × E interaction heritability in BMI. Comparison of results for G × E interaction heritability calculated by LDSC (**a**) and LDAK-SumHer (**b**). The *x*-axis indicates the –log_10_ G × E interaction heritability *P*-value. MET: metabolic equivalent task; TDI: Townsend depriviation index at recruitment; LDSC: linkage disequilibrium score regression; LDAK-SumHer: linkage disequilibrium adjusted kinships–software for estimating SNP heritability from summary statistics.

To confirm these estimates of G × E heritability calculated by LDSC, we used another approach, the LDAK-SumHer v.5.1 method (http://dougspeed.com/ldak/), which is based on a different algorithm from LDSC^39,42^. The heritabilities of G × E interaction calculated using the LDAK-SumHer method are presented in Table 3. Similar to the LDSC results, MET score (*P* = 1.28 × 10^−4^), pack years of smoking (*P* = 3.04 × 10^−5^), and alcohol intake frequency (*P* = 5.15 × 10^−4^) showed statistical significances, based on the Bonferroni multiple correction (*P* < 3.57 × 10^−3^) (Table 3). However, the effects of time spent watching TV (*P* = 1.29 × 10^−2^) and fed-up feelings (*P* = 9.74 × 10^−3^) did not reach a significance level based on the multiple correction in the LDAK-SumHer analysis. The heritability of G × E interaction for alcohol intake frequency was *h*_G×E_^2^ = 0.0032, that for MET score was *h*_G×E_^2^ = 0.0045, and that for pack years of smoking was *h*_G×E_^2^ = 0.0052 in LDAK-SumHer analysis. The *P*-values for the heritability of G × E interaction calculated by LDSC and LDAK-SumHer methods are compared in Fig. 2, which show a similar pattern between them across lifestyle factors.

**Table 3:** G × E interaction heritability calculated using the LDAK-SumHer method.

Category	Lifestyle factor	*N*	G × E interaction heritability % (SE)	*P*-value
Physical activity	MET score^a^	268,536	0.45 (0.12)	1.28 × 10^−4^
	Moderate physical activity	315,747	0.17 (0.10)	4.48 × 10^−2^
Physical activity	Time spent watching television (TV)	328,943	0.23 (0.10)	1.29 × 10^−2^
(Leisure life)	Time spent using computer	328,824	5.00 × 10^−4^ (0.097)	5.00 × 10^−1^
Smoking	Smoking Status	330,138	0.22 (0.10)	1.79 × 10^−2^
	Pack years of smoking^a^	279,758	0.52 (0.13)	3.04 × 10^−5^
Alcohol	Alcohol intake status^b^	330,995	0.14 (0.09)	5.71 × 10^−2^
	Alcohol intake frequency^a,b^	331,049	0.32 (0.10)	5.15 × 10^−4^
Mental health	Neuroticism score	269,392	0.14 (0.12)	1.14 × 10^−1^
	Fed-up feelings	324,753	0.23 (0.098)	9.74 × 10^−3^
Sleep	Sleep duration^b^	329,523	0.14 (0.09)	5.12 × 10^−2^
	Nap during day	331,141	0.13 (0.099)	9.90 × 10^−2^
Social economic status	Average total household income before tax	285,544	0.18 (0.11)	4.48 × 10^−2^
	Townsend deprivation index at recruitment	330,893	0.23 (0.10)	1.16 × 10^−2^

*MET score* metabolic equivalent of task score, *SE* standard error.

^a^Indicates the statistically significant G × E interaction heritability based on the Bonferroni corrected *P*-value threshold (*P*-value < 3.57 × 10^−3^).

^b^Indicates the GWIS after genomic control.

### G × E interaction of MET score, pack years of smoking, and alcohol intake frequency in other obesity-related traits

To investigate whether MET score, pack years of smoking, and alcohol intake frequency also showed statistically significant heritability of G × E interaction in other obesity-related traits, we tested the G × E interaction in waist circumference (WC), hip circumference (HC), waist-to-hip ratio adjusted with BMI (WHRadjBMI), and body fat percentage (BFP). The lifestyle characteristics of participants related to these obesity-related traits are summarized in Supplementary Tables 9–12. Similar to that done when determining BMI, the participants were divided into quartiles based on their trait values, and as expected, the average BMI increased in each successive group for all traits. The mean value of MET score in each quartile group demonstrated that participants with more active lifestyles generally exhibited lower values of those obesity-related traits. Higher pack years of smoking and alcohol intake frequency were generally related to higher BMI values. The MET score, pack years of smoking, and alcohol intake frequency showed a significant association under multiple testing (*P* < 4.16 × 10^−3^) with all obesity-related traits (Supplementary Table 13). Similar to the results for BMI, MET score exhibited negative effect size (*β*) in all obesity-related traits, whereas alcohol intake frequency and pack years of smoking showed positive effect size (*β*) in all obesity-related traits. The distributions of raw and processed variables for obesity-related traits in the population are shown in Supplementary Fig. 4 as histograms.

Further, we examined correlations among these obesity-related traits in the study population (Supplementary Fig. 5). As expected, all traits showed positive correlations with BMI, with strongest correlation seen in HC (correlation coefficient value: 0.86), followed by that in WC (correlation coefficient value: 0.81), BFP (correlation coefficient value: 0.57), and WHRadjBMI (correlation coefficient value: 0.44).

Using the same method as that for BMI analysis, we tested G × E interactions between the 4,143,506 SNPs and MET score, pack years of smoking, and alcohol intake frequency in WC, HC, WHRadjBMI, and BFP adjusted age, sex, genotyping array, and PC1–PC10. To investigate genomic inflation, we estimated the intercept of LD score regression from the each GWIS result in the Supplementary Table 14. Since the intercept values of alcohol intake frequency on WC (1.16), alcohol intake frequency on HC (1.17), and alcohol intake frequency on WHRadjBMI (1.13) were higher than 1.1, we applied the genomic control correction using lambda value to these traits. As a result, the intercept values become decreased after the correction: alcohol intake frequency on WC (0.97), alcohol intake frequency on HC (0.98), and alcohol intake frequency on WHRadjBMI (0.97) (Supplementary Table 14). The GWIS results are shown in Supplementary Figs. 6–8 as Manhattan plots in Supplementary Figs. 9–11 as Q–Q plots. We could not find genome-wide significant signals after multiple correction (*P* < 4.17 × 10^−9^ calculated by 5.00 × 10^−8^/12) for G × E interaction in the MET score and pack years of smoking, while two statistically significant signals (rs5729295 and rs11642015 at the *FTO* locus) were identified in alcohol intake frequency (Supplementary Table 15). Using RegulomeDB and the similar method as that used with BMI, we investigated functional annotation of these two statistical significant (*P* < 4.17 × 10^−9^) SNPs (Supplementary Table 16). Further, we determined any association of genetic variants existing within the 10 Mb flanking region of the lead SNPs with obesity or obesity-related traits (Supplementary Table 17), a direct association of the lead SNPs with any traits using the GWAS catalog (Supplementary Table 18), and the eQTL information using GTEx version 8 (Supplementary Table 19)^41^. We found that two lead SNPs were previously reported to be associated with BMI. These SNPs of the *FTO* locus (rs57292959, and rs11642015) were associated with diverse traits, including BMI, and showed eQTL genes as sentinel SNPs (*r*^2^ = 1).

Additionally, we compared the results of suggestive G × E interactions in BMI (*P* < 5.00 × 10^−6^) with the results in other obesity-related traits (Supplementary Tables 20–23). Notably, rs11642015 that yielded genome-wide significant interaction in BMI (near *FTO* gene, *β* = 8.29E-04, SE = 1.31E-03, *P* = 2.79 × 10^−10^) was also statistically significant for WC (*β* = 1.11E-02, SE = 1.47E-03, *P* = 5.33 × 10^−12^) and BFP (*β* = 9.41E-03, SE = 1.60E-03, *P* = 4.48 × 10^−9^).

**Fig. 3:**
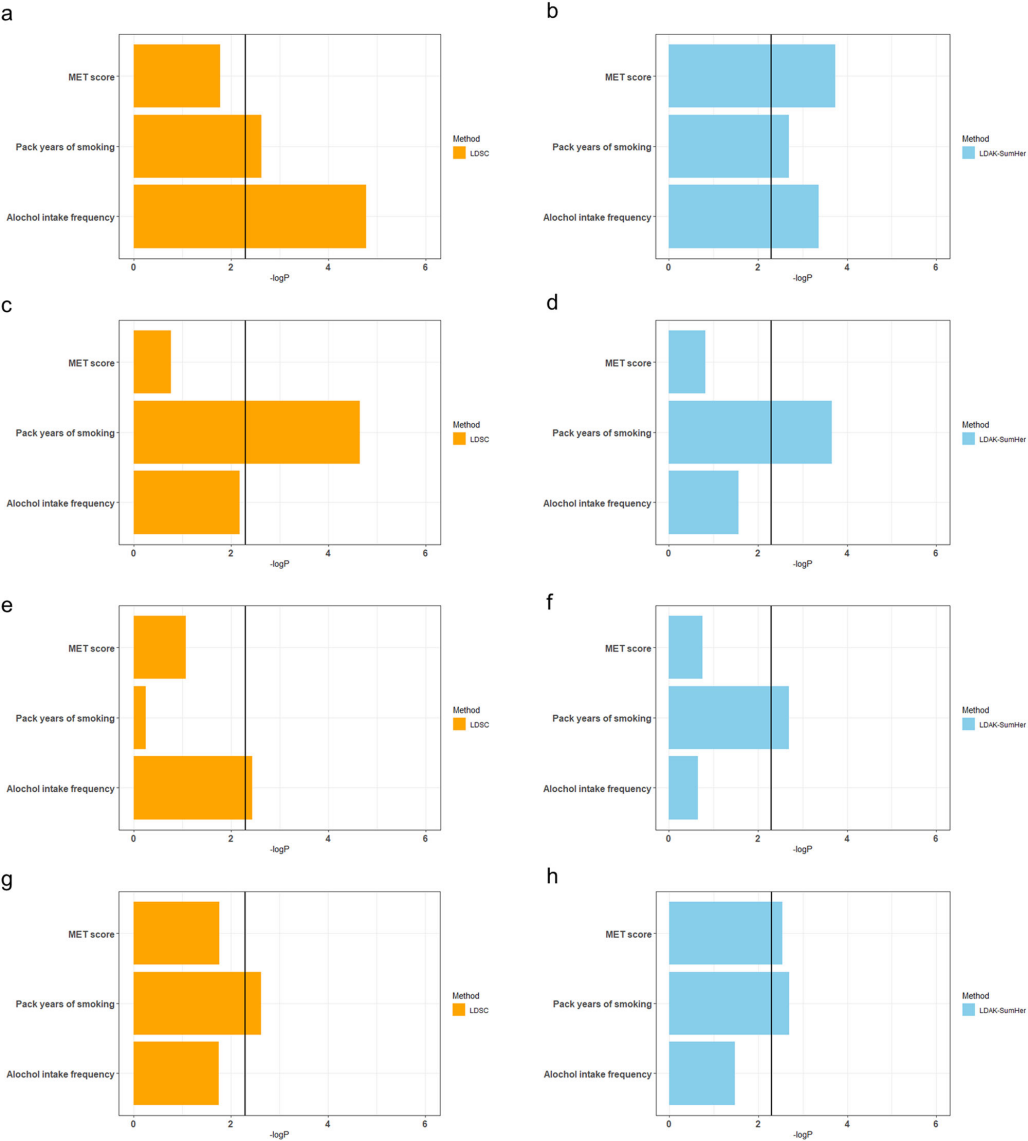
Bar plots of G × E interaction heritability in obesity-related traits. Comparison of results for G × E interaction heritability calculated by LDSC (**a**, **c**, **e**, **g**) and LDAK-SumHer (**b**, **d**, **f**, **h**) in waist circumference (**a**, **b**), hip circumference (**c**, **d**), WHRadjBMI (**e**, **f**), and body fat percentage (**g**, **h**). The *x*-axis indicates the –log_10_ G × E interaction heritability *P*-value. MET metabolic equivalent task, WHRadjBMI waist-to-hip ratio adjusted with body mass index, LDSC linkage disequilibrium score regression, LDAK-SumHer linkage disequilibrium adjusted kinships–software for estimating SNP heritability from summary statistics.

Based on these results, we calculated the heritability of G × E interaction for MET score, pack years of smoking, and alcohol intake frequency using LDSC and LDAK-SumHer for each trait (Fig. 3 and Tables 4–5). Using the Bonferroni multiple correction (*P* < 4.17 × 10^−3^), the heritabilities of G × E interaction for pack years of smoking in WC (*h*_G×E_^2^ = 0.0065, *P* = 2.36 × 10^−3^), alcohol intake frequency in WC (*h*_G×E_^2^ = 0.0083, *P* = 1.66 × 10^−5^), pack years of smoking in HC (*h*_G×E_^2^ = 0.0102, *P* = 2.25 × 10^−5^), and alcohol intake frequency in WHRadjBMI (*h*_G×E_^2^ = 0.0059, *P* = 3.66 × 10^−3^) were statistically significant according to the LDSC analysis. In case of LDAK-SumHer analysis, the heritabilities of G × E interaction for MET score in WC (*h*_G×E_^2^ = 0.0044, *P* = 1.84 × 10^−4^), pack years of smoking in WC (*h*_G×E_^2^ = 0.0034, *P* = 2.00 × 10^−3^), alcohol intake frequency in WC (*h*_G×E_^2^ = 0.0039, *P* = 4.34 × 10^−4^), pack years of smoking in HC (*h*_G×E_^2^ = 0.0043, *P* = 3.20 × 10^−4^), and MET score in BFP (*h*_G×E_^2^ = 0.0033, *P* = 2.88 × 10^−3^) were statistically significant.

**Table 4:** G × E interaction heritability calculated using the LDSC method.

G × E interaction heritability					
Phenotype	Lifestyle factor	*N*	G × E interaction heritability % (SE)	*P*-value	Intercept
Waist circumference	MET score	268,484	0.51 (0.24)	1.68 × 10^−2^	1.06
	Pack years of smoking^a^	279,704	0.65 (0.23)	2.36 × 10^−3^	1.03
	Alcohol intake frequency^a,b^	330,986	0.70 (0.16)	6.07 × 10^−6^	0.97
Hip circumference	MET score	268,487	0.23 (0.24)	1.69 × 10^−1^	1.07
	Pack years of smoking^a^	279,707	1.02 (0.25)	2.25 × 10^−5^	1.05
	Alcohol intake frequency^a,b^	330,994	0.51 (0.17)	1.35 × 10^−3^	0.98
WHRadjBMI	MET score	268,465	0.29 (0.21)	8.36 × 10^−2^	1.07
	Pack years of smoking	279,684	X	-	1.02
	Alcohol intake frequency^b^	330,965	0.43 (0.19)	1.18 × 10^−2^	0.97
Body fat percentage	MET score	264,667	0.53 (0.25)	1.70 × 10^−2^	1.03
	Pack years of smoking	275,885	0.42 (0.21)	2.28 × 10^−2^	1.03
	Alcohol intake frequency	326,077	0.40 (0.19)	1.76 × 10^−2^	1.09

*MET score* metabolic equivalent of task score, *WHRadjBMI* waist to hip ratio adjusted for body mass index, *SE* standard error.

^a^Indicates the statistically significant G × E interaction heritability based on the Bonferroni corrected *P*-value threshold (*P*-value < 4.17 × 10^−3^).

^b^Indicates the GWIS after genomic control.

**Table 5:** G × E interaction heritability calculated using the LDAK-Sum Her method.

G × E interaction heritability				
Phenotype	Lifestyle factor	*N*	G × E interaction heritability % (SE)	*P*-value
Waist circumference	MET score^a^	268,484	0.44 (0.12)	1.84 × 10^−4^
	Pack years of smoking^a^	279,704	0.34 (0.12)	2.00 × 10^−3^
	Alcohol intake frequency^a,b^	330,986	0.33 (0.10)	3.88 × 10^−4^
Hip circumference	MET score	268,487	0.13 (0.12)	1.50 × 10^−1^
	Pack years of smoking^a^	279,707	0.43 (0.13)	3.20 × 10^−4^
	Alcohol intake frequency^b^	330,994	0.11 (0.10)	1.30 × 10^−1^
WHRadjBMI	MET score	268,465	0.12 (0.12)	1.74 × 10^−1^
	Pack years of smoking	279,684	0.03 (0.10)	3.85 × 10^−1^
	Alcohol intake frequency^b^	330,965	0.07 (0.09)	2.16 × 10^−1^
Body fat percentage	MET score^a^	264,667	0.33 (0.12)	2.88 × 10^−3^
	Pack years of smoking	275,885	0.24 (0.12)	2.07 × 10^−2^
	Alcohol intake frequency	326,077	0.19 (0.10)	3.34 × 10^−2^

*MET score* metabolic equivalent of task score, *WHRadjBMI* waist to hip ratio adjusted for body mass index, *SE* standard error.

^a^Indicates the statistically significant G × E interaction heritability based on the Bonferroni corrected *P*-value threshold (*P*-value < 4.17 × 10^−3^).

^b^Indicates the GWIS after genomic control.

## Discussion

In this study, to estimate the proportion of missing heritability that could be explained by G × E interaction, we determined the heritability of G × E interaction for BMI and other obesity-related traits in a large sample of 331,282 participants from the UK Biobank. Three lifestyle factors—MET score, pack years of smoking, and alcohol intake frequency—showed statistically significant interaction with genetic factors for BMI in both LDSC and LDAK-SumHer analyses. The G × E interaction heritability (%) and standard error of these factors by LDSC and LDAK-SumHer were as follows: MET score, 0.45% (0.12) and 0.65% (0.24); pack years of smoking, 0.52% (0.13) and 0.93% (0.26); and alcohol intake frequency, 0.32% (0.10) and 0.80% (0.17), respectively. Moreover, we identified the statistical significance of the G × E interaction heritability of these three lifestyle factors in WC, HC, WHRadjBMI, and BFP. Additionally, we identified two genome-wide significant loci interacting with lifestyle factors in these obesity-related traits.

Recently, Rask-Andersen et al.^29^ and Tyrrell et al.^28^ calculated the PRS for BMI in the European sample referring to SNPs discovered by Locke et al.^43^. They investigated the interactions between the PRS and lifestyle factors using the linear regression model. Rask-Andersen et al. found physical activity, alcohol intake frequency, and socioeconomic status to interact with PRS in BMI. Tyrrell et al. found physical activity and socioeconomic status to interact with PRS in BMI. Similarly, we also found a statistical significance in the G × E interaction heritability of physical activity (MET score) and alcohol intake frequency in BMI. While smoking (pack years of smoking) was found to be significant only in this study, Robinson et al. previously demonstrated the significance of G × E interaction smoking interaction heritability^34^.

Robinson et al. proposed the heritability of G × E interaction by estimating the difference in heritabilities between subgroups classified by environmental exposure using mixed-effect models^36^. They found evidence for the contribution of G × E interaction for smoking behavior to BMI, which explains 4.0% of the phenotypic variance. Our analysis estimated statistically significant heritability of G × E interaction in the pack years of smoking in BMI with somewhat less value (*h*_G×E_^2^ = 0.93%, *P* = 1.74 × 10^−4^ calculated by LDSC; *h*_G×E_^2^ = 0.52%, *P* = 3.04 × 10^−5^ calculated by LDAK-SumHer). Sulc et al. also provided evidence for G × E interaction effect on BMI based on the calculation of phenotypic variance across the different PRS groups and found PRS × E to contribute 1.9% to BMI^37^. If we assume that there is no correlation between G × E interaction of three lifestyle factors, the G × E interaction effect on BMI may be calculation by the summation of the heritabilities of three lifestyle factors. The summed values account to 1.3% for LDAK-SumHer and 2.38% for LDSC in this study. Sulc et al. reported TDI and alcohol intake frequency as lifestyle factors for the PRS × E contribution of 1.9% to BMI. We also found marginally significant heritability for G × E interaction in TDI (*h*_G×E_^2^ = 0.42%, *P* = 6.70 × 10^−3^ calculated by LDSC; *h*_G×E_^2^ = 0.23%, *P* = 1.16 × 10^−2^ calculated by LDAK-SumHer). And Shin et al. estimated heritability of G × E interaction in BMI using the GxEsum program, which was built on LDSC approach^44^. The GxEsum is a method for estimating the phenotypic variance explained by genome-wide G x E terms for large-scale biobank dataset. They provided the heritability of G × E interaction for age (*h*_G×E_^2^ = 0.4%, *P* = 0.019), neuroticism score (*h*_G×E_^2^ = 0.7%, *P* = 1.61 × 10^−5^), physical activity (*h*_G×E_^2^ = 0.3% *P* = 0.026) and alcohol intake frequency (*h*_G×E_^2^ = 0.3%, *P* = 0.060) in BMI. Moreover, we also found marginally significant heritability for G × E interaction using the LDSC method as follows: neuroticism score (*h*_G×E_^2^ = 0.52%, *P* = 9.05 × 10^−3^), MET score (*h*_G×E_^2^ = 0.65% *P* = 3.38 × 10^−3^) and alcohol intake frequency (*h*_G×E_^2^ = 0.80%, *P* = 1.26 × 10^−6^).

Our analysis for the G × E interaction found two genome-wide significant loci for multiple testing (*P* < 3.57 × 10^−9^ on BMI, *P* < 4.17 × 10^−9^ on obesity-related traits) (Supplementary Tables 3 and  15). All two loci have been reported to be associated with BMI (Supplementary Tables 6 and  17). Notably, two loci were previously reported for their interactions with lifestyle factors in BMI, supporting the validity of our G × E interaction analysis. First, the locus of the *CHRNA* (cholinergic receptor nicotinic alpha subunit) gene cluster on chromosome 15 has been well-reported to be associated with smoking addiction^45–48^. For rs12438181, which showed significant effects on BMI by interacting with smoking behaviors in this study, eQTL analysis of GTEx (ver. 8) data revealed that this SNP is associated with both *CHRNA3* and *CHRNA5* genes in the brain (Supplementary Table 8)^41^. Further, two recent studies have provided evidence for the interaction between *CHRNA3-A5-B4* gene cluster variants and smoking behavior in BMI^35,49^. Taylor et al. suggested that CHRNAs modulate responses such as food appetite to rewarding stimuli, including smoking^49^. The *FTO* (fat mass and obesity associated-alpha-ketoglutarate dependent dioxygenase) gene is a well-known, strong genetic factor for obesity, of which the mechanism is mainly attributable to the role in energy metabolism^50^. Previous studies demonstrated that the *FTO* gene is not only associated with BMI, but also interacts with lifestyle factors to influence BMI^24,26,31^. In this study, rs11642015, and rs57292959 at the *FTO* locus interacted with alcohol intake frequency to affect BMI, WC, and BFP, respectively (Supplementary Tables 3 and  15). Moreover, GTEx (ver. 8) analysis of these SNPs showed the *FTO* gene as an eQTL gene in the skeletal muscle and pancreas (Supplementary Tables 8 and  19)^41^. Further, it was reported that *FTO* [rs1421085 (*r*^2^ = 0.95 from rs11642015)] interacts with the frequency of alcohol consumption in BMI, which supports our findings on the interaction of these lead SNPs with alcohol intake frequency^24^.

There are several limitations to our study. First, some of the GWIS results may be statistically inflated. This can be inferred from the Q–Q plot and the intercept values calculated through the LDSC (Supplementary Fig. 3 and Table 2)^38^. This statistical inflation can occur because of trait polygenicity and large sample size in the study^51,52^. Therefore, these findings await replication and more careful testing with a larger GWAS data. Second, to estimate the GxE interaction heritability, we used LDSC and LDAK-SumHer methods. Both methods were optimized to calculate genetic heritability using the summary statistics estimated fixed effects of linear regression. However, although GWIS is calculated as fixed effects of linear regression model, it is necessary to validate that G × E interaction heritability is estimated using GWIS simulated with various cases. Third, we simply summed the heritabilities of G × E interaction for individual lifestyle factors to estimate total G × E interaction heritability as 1.29–2.38% in BMI. If these heritabilities of different lifestyle factors are dependent on each other, the summed heritability for BMI may be lower than 1.35–2.55%. However, Sulc et al. reported 1.9% total heritability for the contribution of PRS × E interaction to the phenotypic variance of BMI^35^. Moreover, the heritabilities of G × E interaction for other obesity-related traits analyzed in this study were not much different from these. The *h*_G×E_^2^ of WC was 1.35% (LDSC) and 0.67% (LDAK-SumHer), and *h*_G×E_^2^ of HC was 1.02% (LDSC) and 0.43% (LDAK-SumHer), indicating that if our results are inflated, the inflation may be small.

In summary, we performed GWIS for BMI using 331,282 participants in the UK Biobank, and calculated the heritability of G × E interaction for 14 lifestyle factors. Among the lifestyle variables, MET score, pack years of smoking, and alcohol intake frequency consistently showed statistically significant G × E interaction heritability for BMI. Our results suggest that apart of the missing heritability in BMI may be explained by the G × E interaction, indicating that consideration of G × E interaction could improve the accuracy of predicting obesity genetically.

## Methods

### UK Biobank resource

We used the UK Biobank database, which is a population‐based database that recruited more than 487,409 individuals aged 40–69 years during 2006–10^53^. For quality control of the sample, we used Neale lab filters (https://github.com/Nealelab/UK_Biobank_GWAS). The sample filters are as follows: PCA calculation filter for selection of unrelated samples; sex chromosome filter for aneuploidy removal; filter of principal components (PCs) for European sample selection to determine British ancestry; and filters for selection of self-reported “White-British”, “Irish”, and “White”. In addition, we selected samples based on the filter for self-reported “White-British”, remaining the final sample as 331,282.

### Ethics approval and consent to participate

All participants provided signed consent to participate in the UK Biobank^54^. UK Biobank has been given ethical approval to collect participant data by the North West Multicentre Research Ethics Committee, the National Information Governance Board for Health & Social Care, and the Community Health Index Advisory Group.

### Genotype data

Baseline genetic imputation data of 93,095,623 SNPs were available in 487,409 participants. UK Biobank participants used the UK Biobank Axiom Array and the UK BiLEVE Axiom Array from Affymetrix (Santa Clara, CA)^55^. Genotyping imputation was performed using UK10K Project and 1000 Genome Project Phase 3 reference panel. We performed quality control analysis using PLINK v.1.90^56^, based on the following exclusion criteria: SNPs with missing genotype call rates >  0.05, MAF < 0.05, and Hardy–Weinberg equilibrium *P*-value < 1.00 × 10^−6^. We excluded SNPs with MAF smaller than 0.05 to avoid potential false-positive results due to the coincidence of a low-frequency variant^31^. Consequently, 4,143,506 SNPs were retained for further analysis.

### Phenotype data

Participants’ weights were assessed using various methods during the initial UK Biobank assessment center visit. Additionally, standing height was measured on a SECA 240 Height Measure. For BMI, we used data-field 21001, which is constructed from height and weight measured. For this study, BMI was transformed using log transformation.

The lifestyle factors used in GWIS were selected based on previous studies on obesity^24,29,40^. The 14 lifestyle factors were metabolic equivalent task score (MET score; field ID: 22040), moderate physical activity (field ID: 884), time spent using computer (field ID: 1080), time spent watching TV (field ID: 1070), neuroticism score (field ID: 20127), fed-up feelings (field ID: 1960), smoking status (field ID: 20116), pack years of smoking (field ID: 20161), alcohol intake frequency (field ID: 1558), alcohol intake status (field ID: 20117), sleep duration (field ID: 1160), nap during day (field ID: 1190), average total household income before tax (field ID: 738), and TDI (field ID: 189) (Table 1). For the GWIS, lifestyle factors (MET score, time spent using computer, time spent watching TV, pack years of smoking, neuroticism score, sleep duration) were transformed to normal distribution using Gaussian function in structured linear mixed model v.0.3.1 (Struct-LMM) (Supplementary Fig. 1)^26^. For moderate physical activity analysis, participants were divided into four groups. For smoking analysis, participants were divided into two groups, one with or without previous history of smoking and the other with current smoking^40^.

### Statistics and reproducibility

The G × E interaction analysis for the 14 lifestyle factors on BMI as a continuous trait was performed fixed effect model of linear regression using the PLINK v.1.90^56^. Also, we analyzed each obesity-related trait as a continuous trait in the same methods. The formula of the linear regression model is such that:

1 $$\begin{array}{c}{{{{{\rm{Phenotype}}}}}}={\beta }_{0}+{\beta }_{1}{{{{{\rm{G}}}}}}+{\beta }_{2}{{{{{\rm{G}}}}}}\times {{{{{\rm{E}}}}}}+{\beta }_{3}{{{{{\rm{E}}}}}}+{\beta }_{4}{{{{{\rm{age}}}}}}+{\beta }_{5}{{{{{\rm{sex}}}}}}+{\beta }_{6}{{{{{\rm{array}}}}}}+{\beta }_{7}{{{{{\rm{PC}}}}}}1+{\beta }_{8}{{{{{\rm{PC}}}}}}2 \,\\ +{\beta }_{9}{{{{{\rm{PC3}}}}}}+{\beta }_{10}{{{{{\rm{PC4}}}}}}+{\beta }_{11}{{{{{\rm{PC5}}}}}}+{\beta }_{12}{{{{{\rm{PC}}}}}}6+{\beta }_{13}{{{{{\rm{PC}}}}}}7+{\beta }_{14}{{{{{\rm{PC}}}}}}8+{\beta }_{15}{{{{{\rm{PC}}}}}}9+{\beta }_{16}{{{{{\rm{PC}}}}}}10+\varepsilon \end{array}$$

*β*_1_ denote the effect size of genotype *β*_2_  denote the effect size of G × E interaction *β*_3_ denote the effect size of lifestyle factor *β*_4_  denote the effect size of age *β*_5_ denote the effect size of sex *β*_6_ denote the effect size of genotyping array *β*_7_ denote the effect size of PC1 *β*_8_ denote the effect size of PC2 *β*_9_ denote the effect size of PC3 *β*_10_ denote the effect size of PC4 *β*_11_ denote the effect size of PC5 *β*_12_ denote the effect size of PC6 *β*_13_ denote the effect size of PC7 *β*_14_ denote the effect size of PC8 *β*_15_ denote the effect size of PC9 *β*_16_ denote the effect size of PC10

To stabilize the genomic inflation, we applied the genomic control to GWIS results showing an intercept value of LD score regression higher than 1.1 (Supplementary Tables 2 and14). For this purpose, firstly, we estimated the median chi-square distribution of the GWIS result. Next, the lambda value was estimated by the median of chi-square divided by 0.45656^57^ Finally, each chi-square of SNP is divided by the lambda value^58^.

We used PLINK to identify independent SNPs. We used LD clumping to retain the most strongly associated SNPs in each region (PLINK v.1.90 –clump-p1 5e-06 –clump-p2 5e-06 –clump-r2 0.01 –clump-kb 1000) in GWIS analysis.

We used LDSC^38^ and LDAK-SumHer^39^ to estimate the G × E interaction heritability from the 14 GWIS summary statistics. The statistical evaluation method is different between two methods. LDSC methods estimates genetic heritability using a regression model, whereas LDAK-SumHer methods calculated genetic heritability using a likelihood model. When using LDSC to estimate G × E interaction heritability, it is necessary to LD score of SNP. Bulik-Sullivan et al. estimated the 1,217,312 SNPs of LD score based on the European 1000 Genomes database and Hap-Map3 SNPs (https://github.com/bulik/ldsc)^38^. We also used LD score to estimate G × E interaction heritability. When using LDAK-SumHer, it is required for well-imputed common SNPs panel. So, we remained SNP satisfied criteria as follows^41^: (1) 1000 Genomes imputation database, (2) Non-ambiguous SNPs, (3) SNPs not in MHC region (http://dougspeed.com/)^39^. Based on these SNPs, we used GCTA model of LDAK-SumHer to estimate the G × E interaction heritability^39^.

We created Manhattan plots, histograms, Q–Q plots, bar plots, and correlograms and performed association and correlation analysis in R version 4.0.3 (www.r-project.org). We used qqman package for Manhattan plots and corrplot package for calculating correlation coefficients and plotting the correlogram. The ggplot2 package was used for plotting bar plots and lme4 package was used for association analysis.

### Investigation of the biological function of significant loci

To investigate the biological function and possible effects of significant variants on various traits, we investigated this information on the GWAS catalog, RegulomeDB, and GTEx version 8. For GWAS catalog, we determined any association of genetic variants existing within the 10 Mb flanking region of the lead SNPs with obesity or obesity-related traits, a direct association of the lead SNPs with any traits using the GWAS catalog^59^ (https://www.ebi.ac.uk/gwas/). We investigated the eQTL information for lead SNPs shown statistical significance using GTEx version 8 database^41^ (https://gtexportal.org/home/). We searched for functional importance of lead SNPs using RegulomeDB^60^ (https://regulomedb.org/regulome-search/).

### Reporting summary

Further information on research design is available in the Nature Portfolio Reporting Summary linked to this article.

## Supplementary information

Supplementary Information

Reporting Summary

## Data Availability

All data supporting the findings of this study are available within the paper and its supplementary information files. The GWIS summary data are deposited in *Zenodo* (10.5281/zenodo.7647748) and displayed on Manhattan plots. Source data underlying the bar plots presented in the main figure are available as Tables 2–5.
