# Supplementary material for: Gene-environment interaction explains a part of missing heritability in human body mass index
Source: Commun Biol. 2023 Mar 25;6:324. doi: 10.1038/s42003-023-04679-4 (PMC10039928; doi:10.1038/s42003-023-04679-4)
Supplement: Supplementary file 1 — Supplementary Information [file 42003_2023_4679_MOESM1_ESM.pdf]

Trait	Distribution of raw variables	Distribution of processed variables
Body mass index	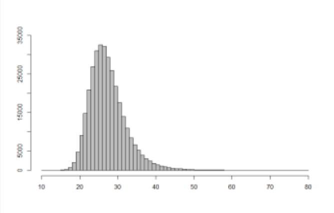	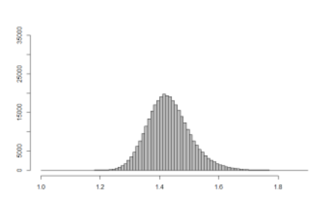
MET score	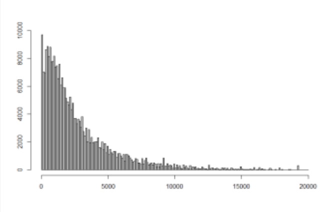	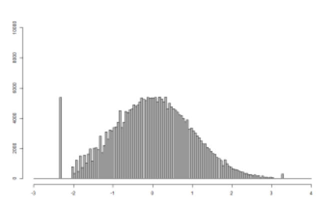
Moderate physical activity	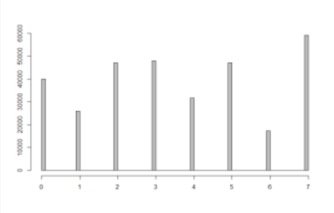	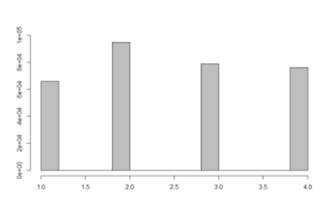
Time spent watching television (TV)	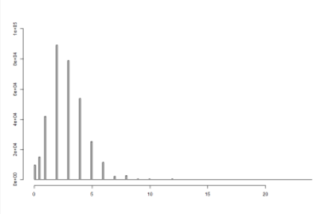	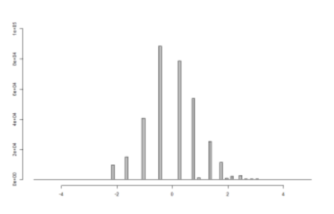
Time spent using computer	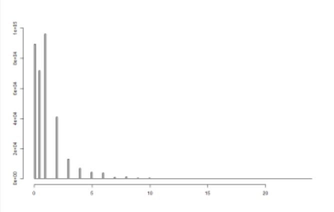	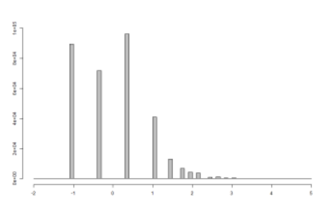
Smoking status	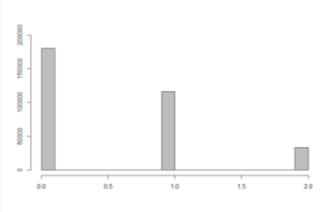	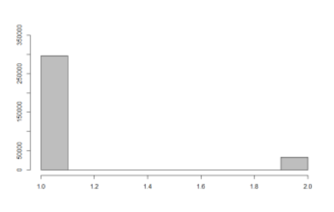
Pack years of smoking	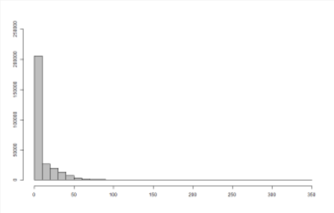	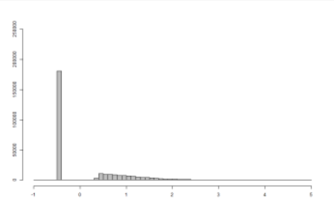

Alcohol intake status	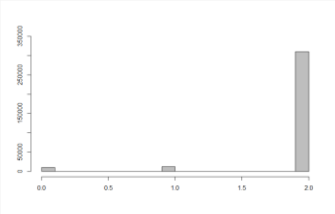	-
Alcohol intake frequency	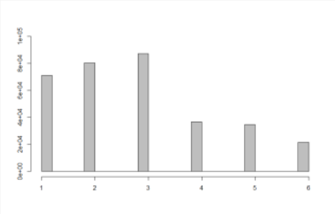	-
Neuroticism score	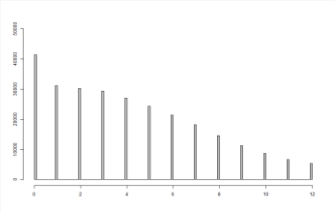	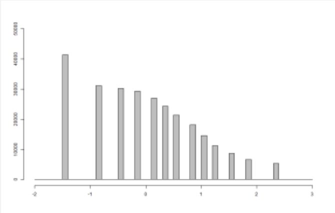
Fed-up feelings	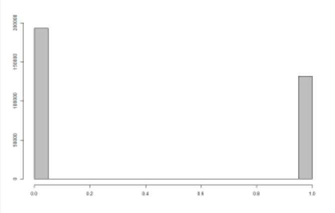	-
Sleep duration	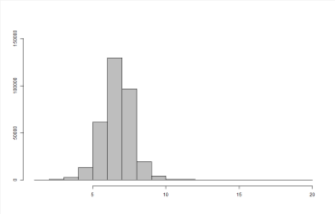	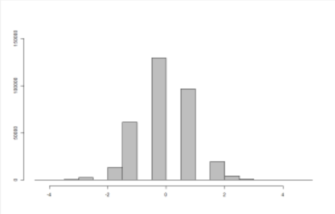
Nap during day	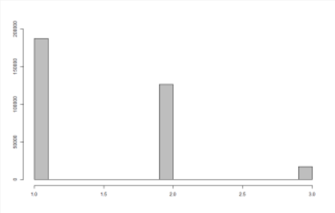	-
Townsend deprivation index at recruitment	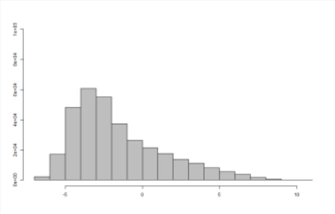	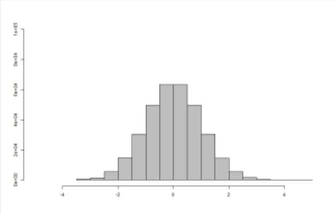

Average total household income before tax	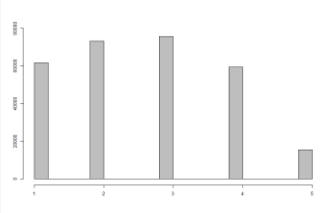	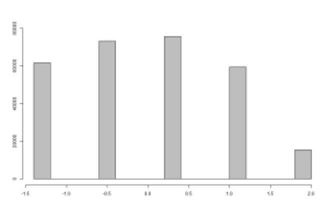

Supplementary Figure 1. Bar plots of 14 obesity-related lifestyles. The distribution of raw variables and processed variables for the 14 obesity-related lifestyles.

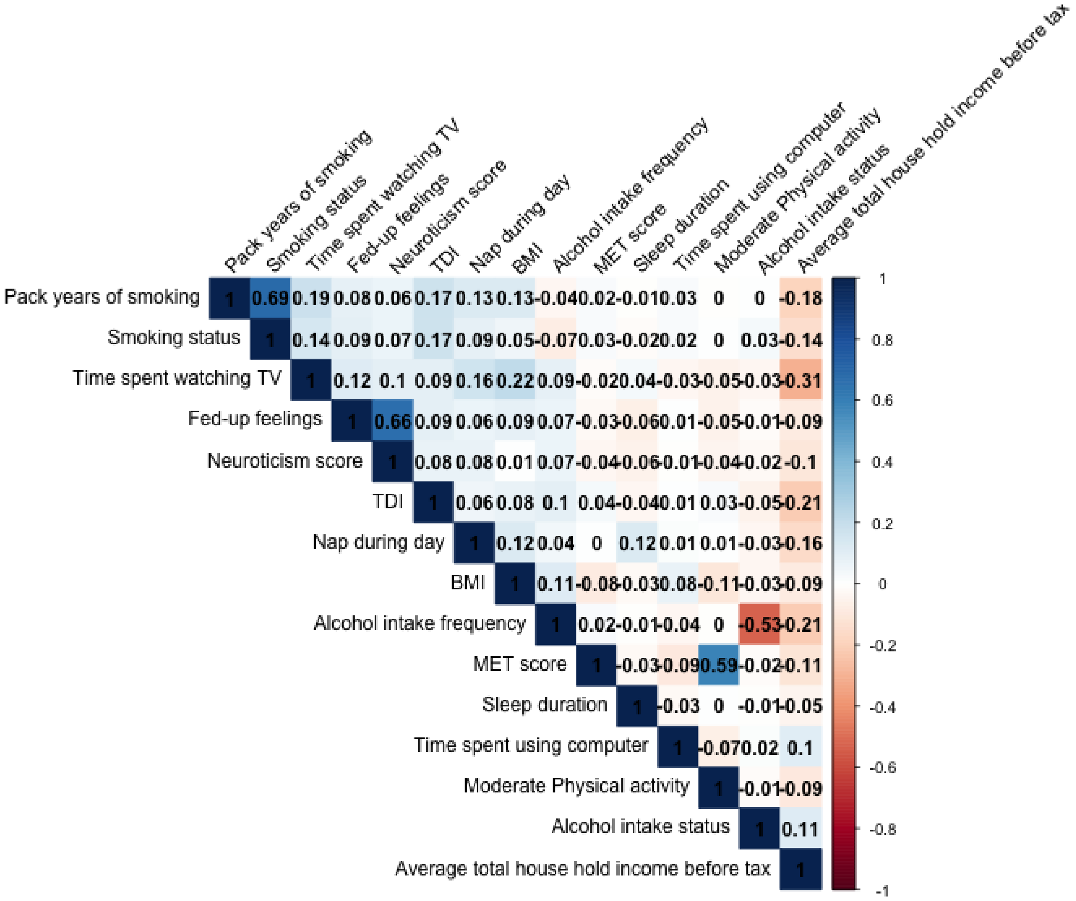

Supplementary Figure 2. Correlogram of between pairs of 14 lifestyle factors and BMI. Correlogram showing the correlation coefficients between pairs of 14 lifestyle factors and between BMI and 14 lifestyle factors.

BMI, body mass index; TDI, Townsend deprivation index at recruitment.

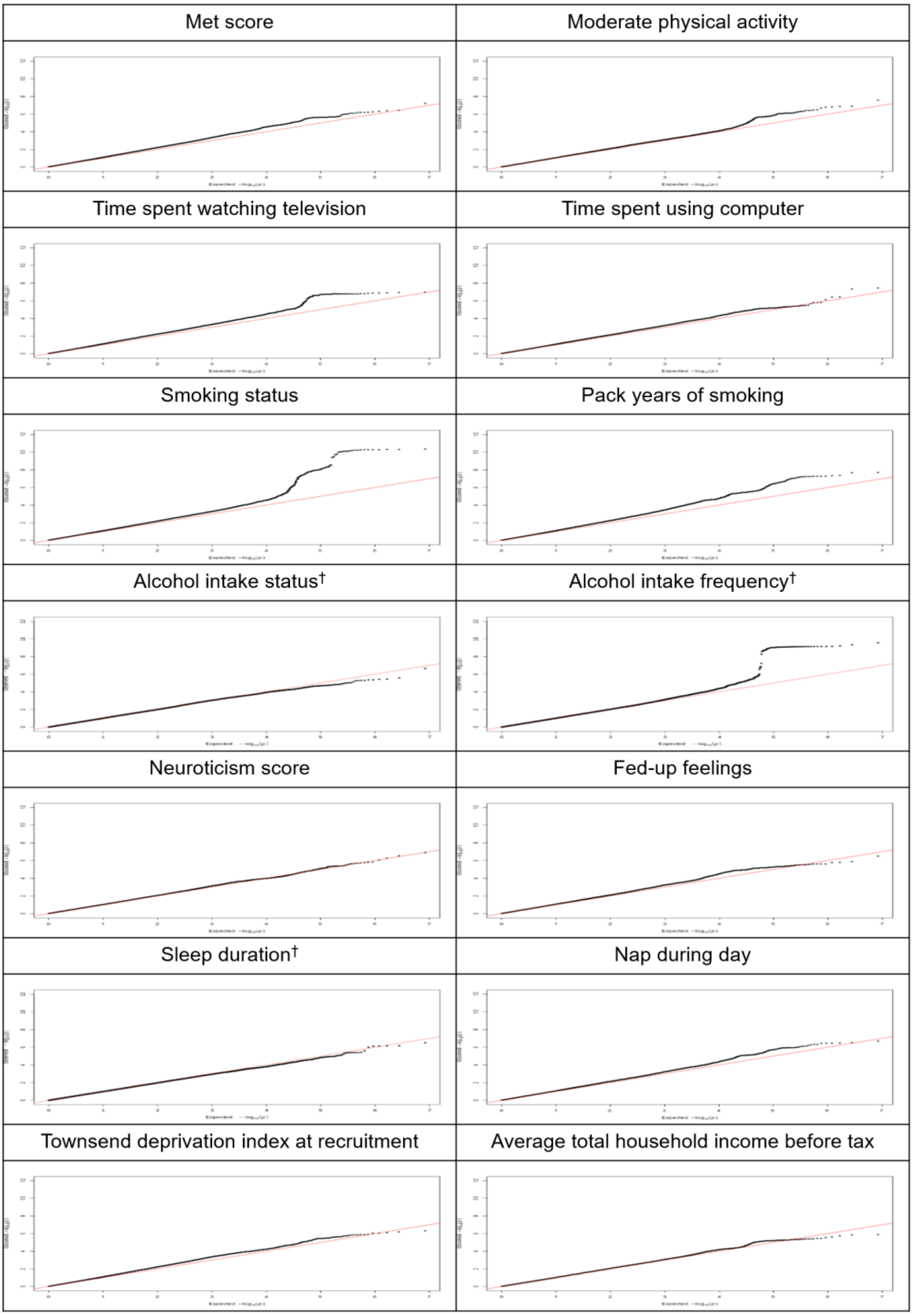

Supplementary Figure 3. Quantile-quantile plots of gene-environment interaction study. Interaction *P*-values between genetic variant and lifestyle factor were computed using Plink v.1.90.

† indicates GWIS after genomic control.

Trait	Distribution of raw phenotype	Distribution of processed phenotype
Waist circumference	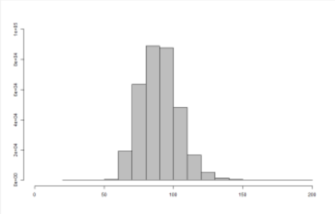	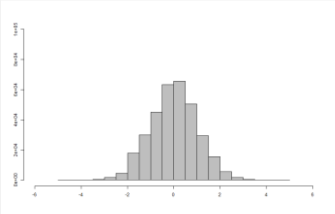
Hip circumference	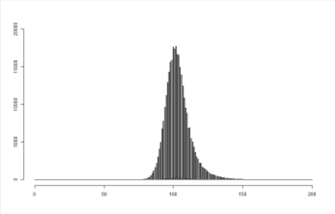	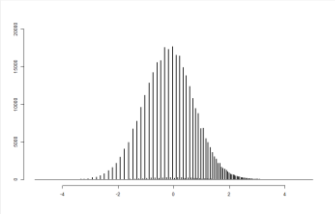
WHRadjBMI	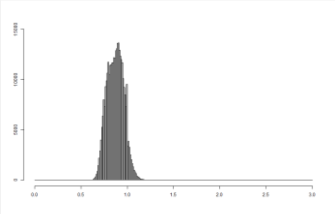	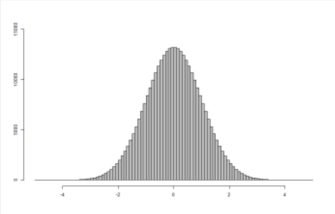
Body fat percentage	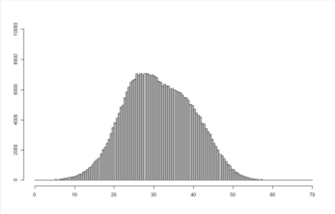	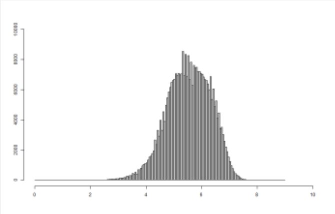

Supplementary Figure 4. Bar plots of obesity-related traits. The distribution of raw variables and processed variables for normalization about the obesity-related traits.

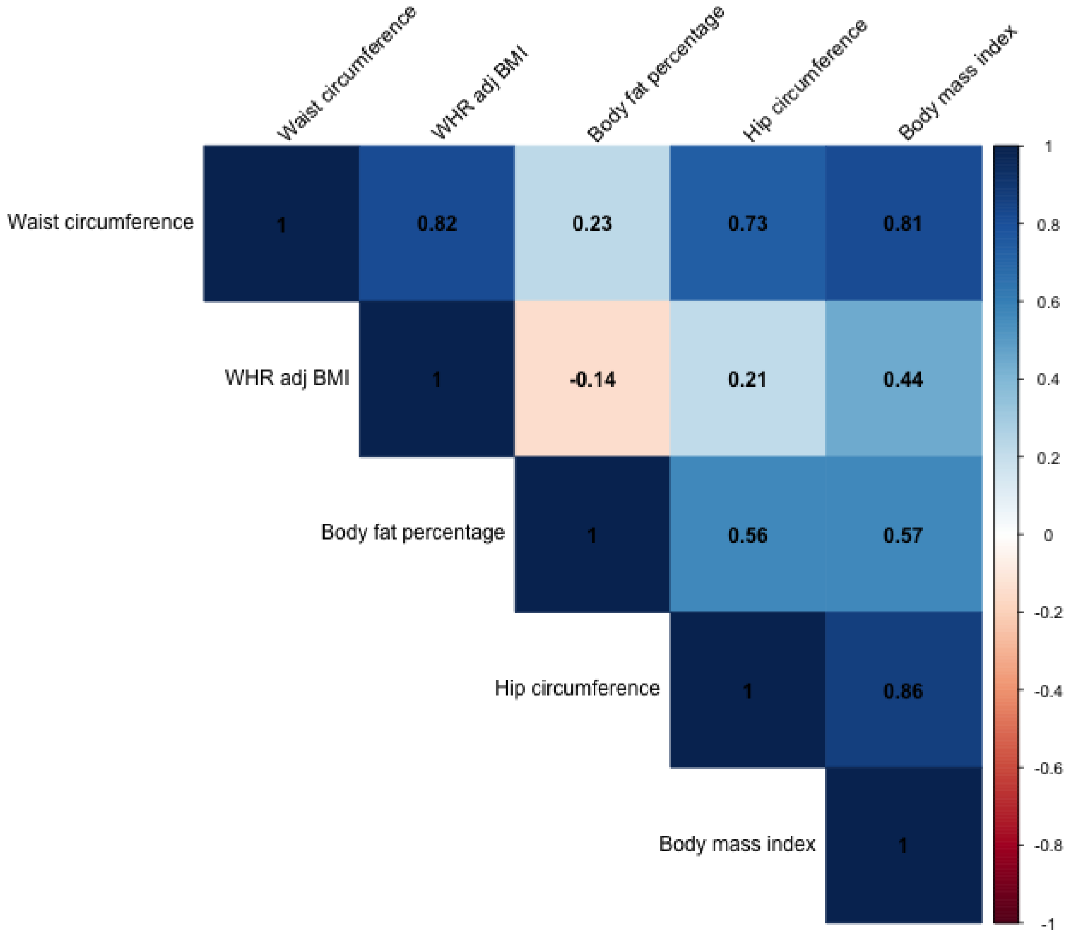

Supplementary Figure 5. Correlogram of between pairs of 14 lifestyle factors and BMI. Correlogram showing the correlation coefficients between pairs of 5 obesity-related traits including BMI.

BMI, body mass index; WHRadjBMI, waist to hip ratio adjusted for body mass index.

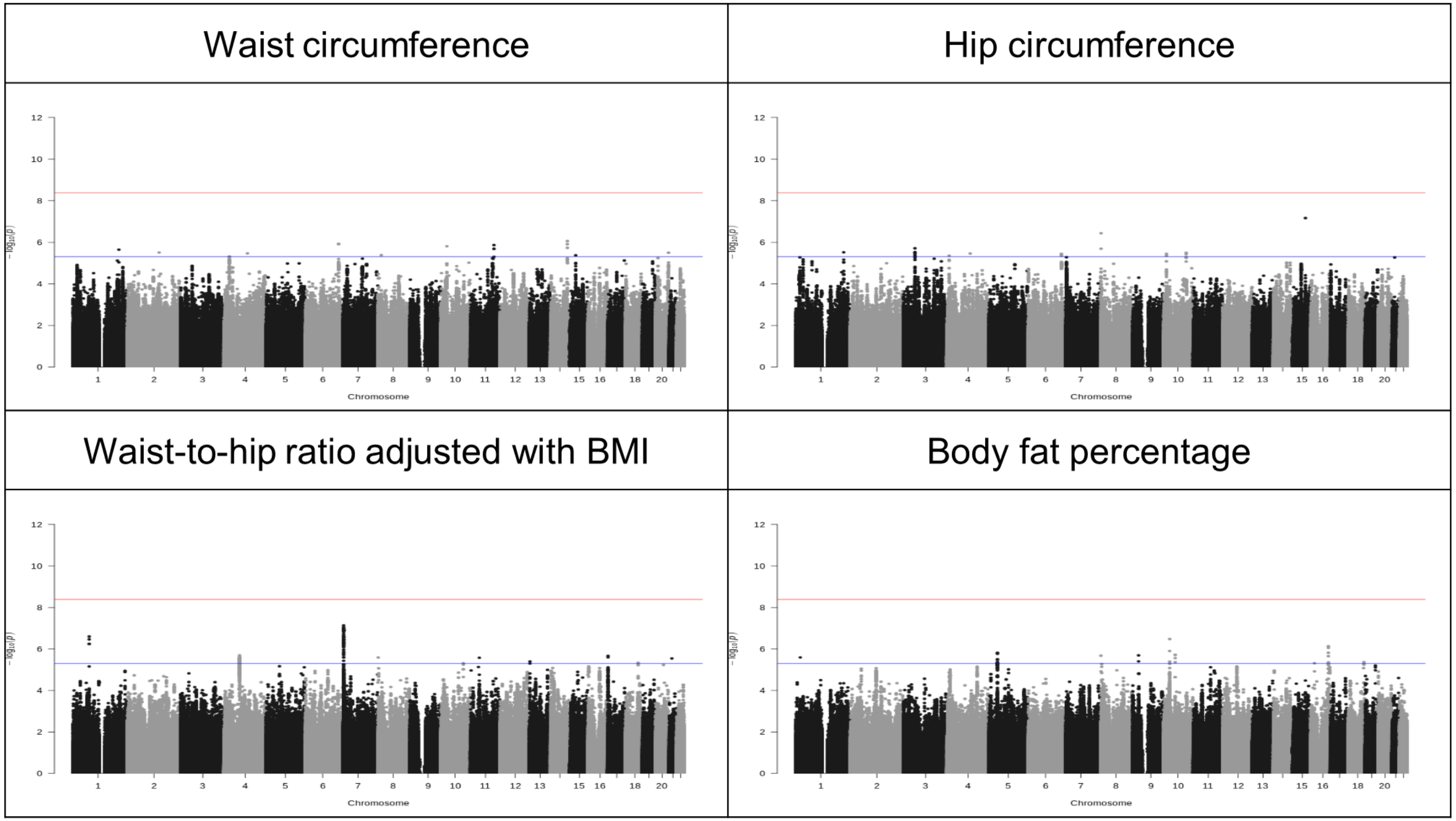

Supplementary Figure 6. Manhattan plots of gene-MET score interaction study in obesity-related traits. Manhattan plot showing the -$\log_{10}$-transformed gene-MET score interaction *P*-value of each SNP on the y axis and base-pair positions along the chromosomes on the x axis. SNP *P*-values were computed in Plink 1.90. The blue line indicates the suggestive threshold ($P < 5.00 \times 10^{-6}$). The red line indicates the genome-wide significance for multiple testing ($P < 4.16{\times}10^{-9}$).

† indicates GWIS after genomic control.

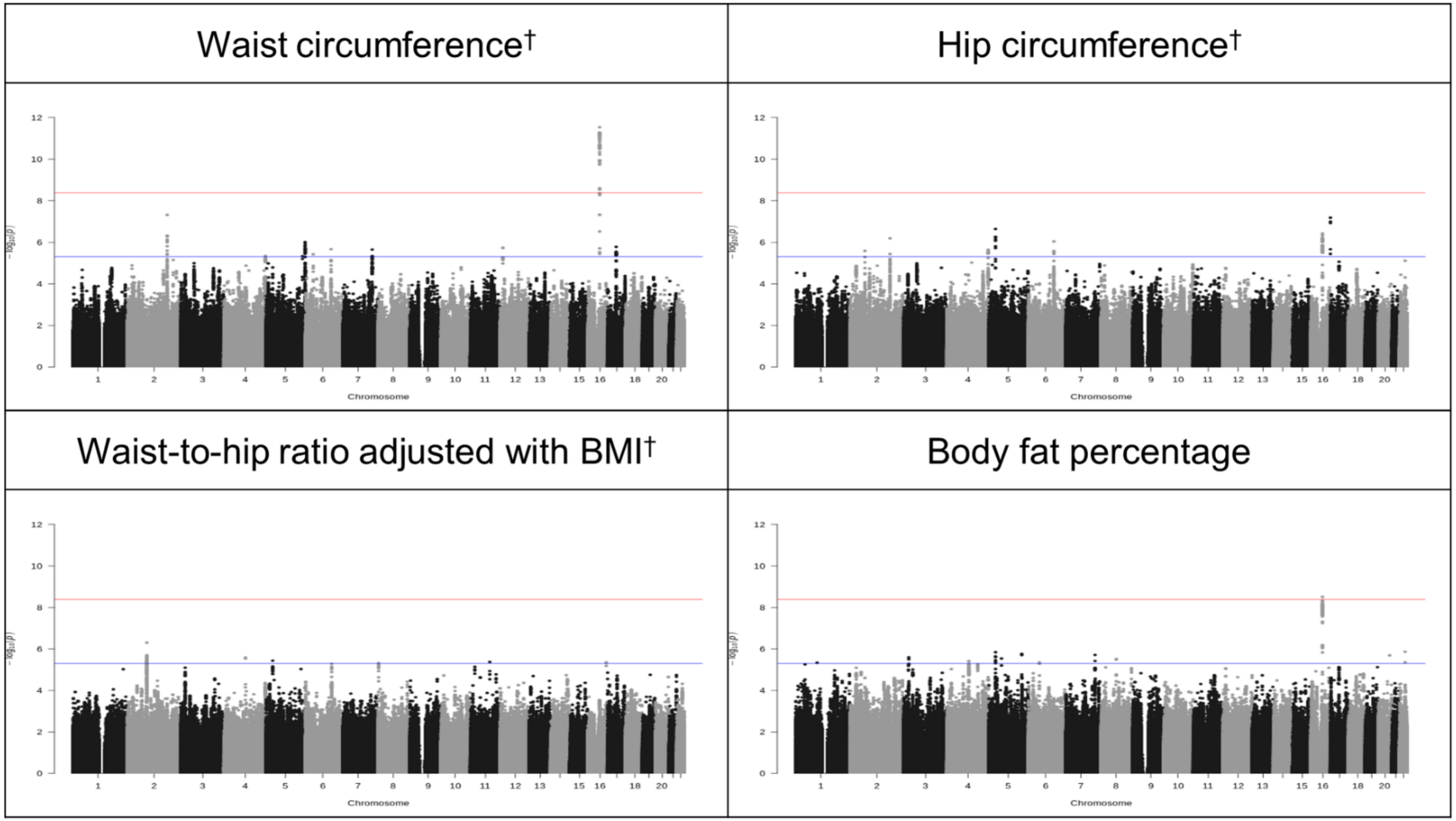

Supplementary Figure 7. Manhattan plots of gene-alcohol intake frequency interaction study in obesity-related traits. Manhattan plot showing the -$\log_{10}$-transformed gene-alcohol intake frequency interaction *P*-value of each SNP on the y axis and base-pair positions along the chromosomes on the x axis. SNP *P*-values were computed in Plink 1.90. The blue line indicates the suggestive threshold ($P < 5.00 \times 10^{-6}$). The red line indicates the genome-wide significance for multiple testing ($P < 4.16\times10^{-9}$).

† indicates GWIS after genomic control.

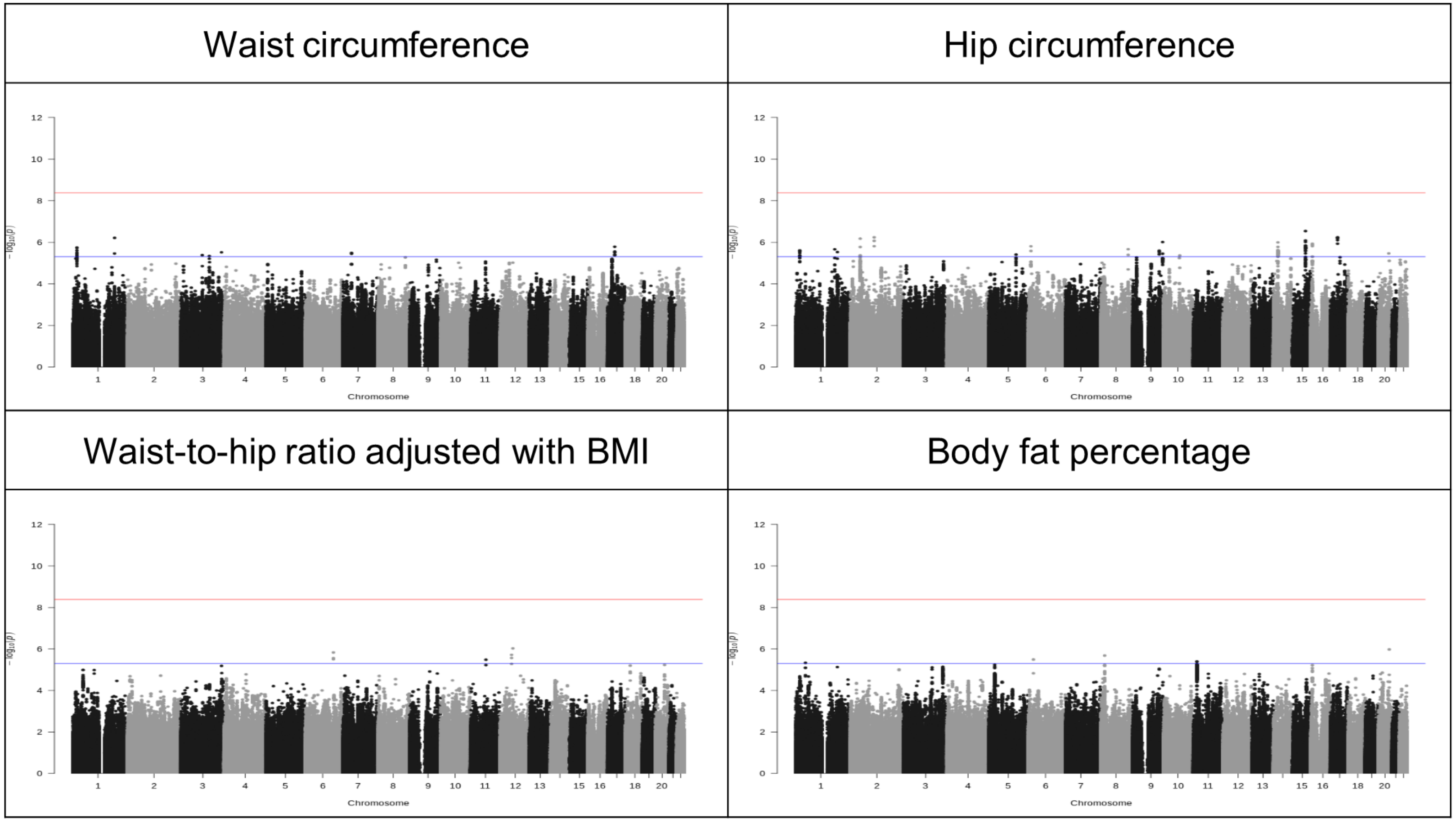

Supplementary Figure 8. Manhattan plots of gene-pack years of smoking interaction study in obesity related traits. Manhattan plot showing the -$\log_{10}$-transformed gene-pack years of smoking interaction *P*-value of each SNP on the y axis and base-pair positions along the chromosomes on the x axis. SNP *P*-values were computed in Plink 1.90. The blue line indicates the suggestive threshold ($P < 5.00 \times 10^{-6}$). The red line indicates the genome-wide significance for multiple testing ($P < 4.16\times10^{-9}$).

† indicates GWIS after genomic control.

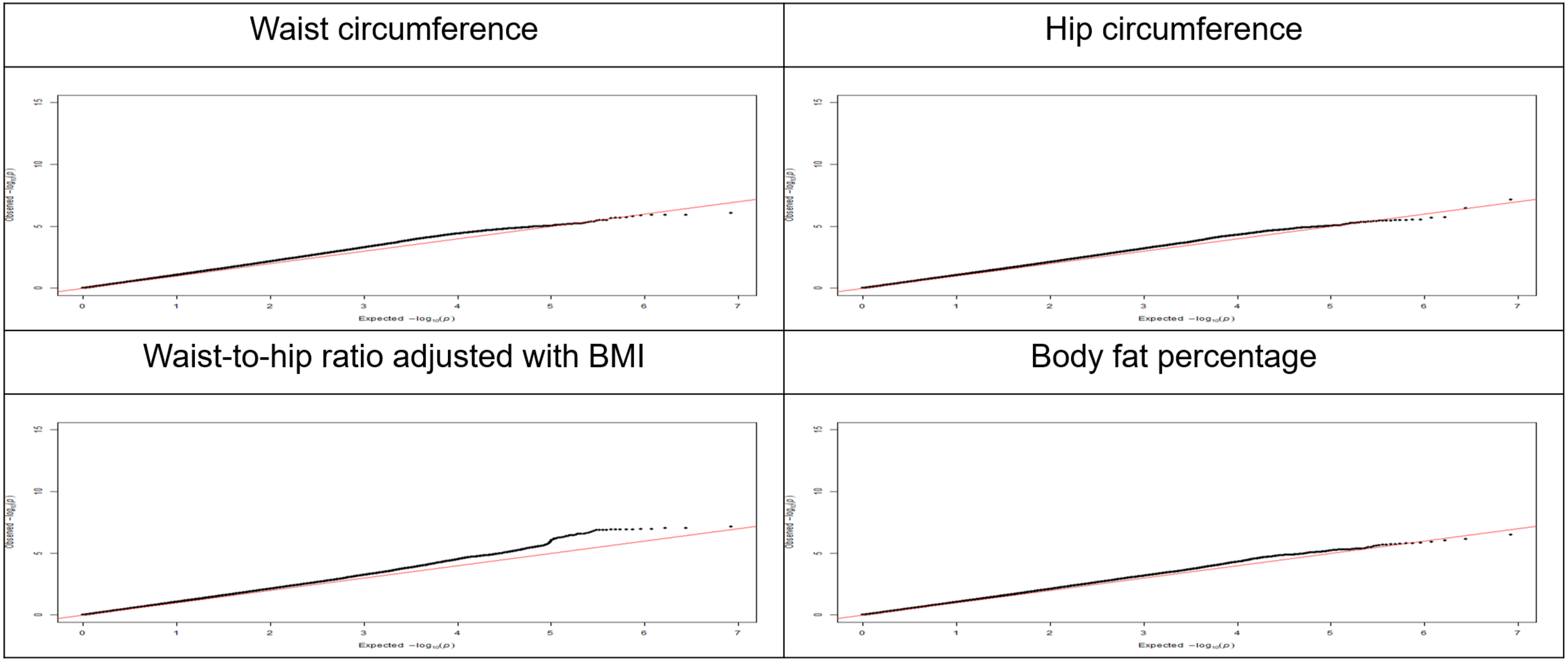

Supplementary Figure 9. Quantile-quantile plot of gene-MET score interaction study in obesity-related traits. Interaction *P*-values between genetic variant and metabolic equivalent of task (MET) score were computed using Plink v.1.90.

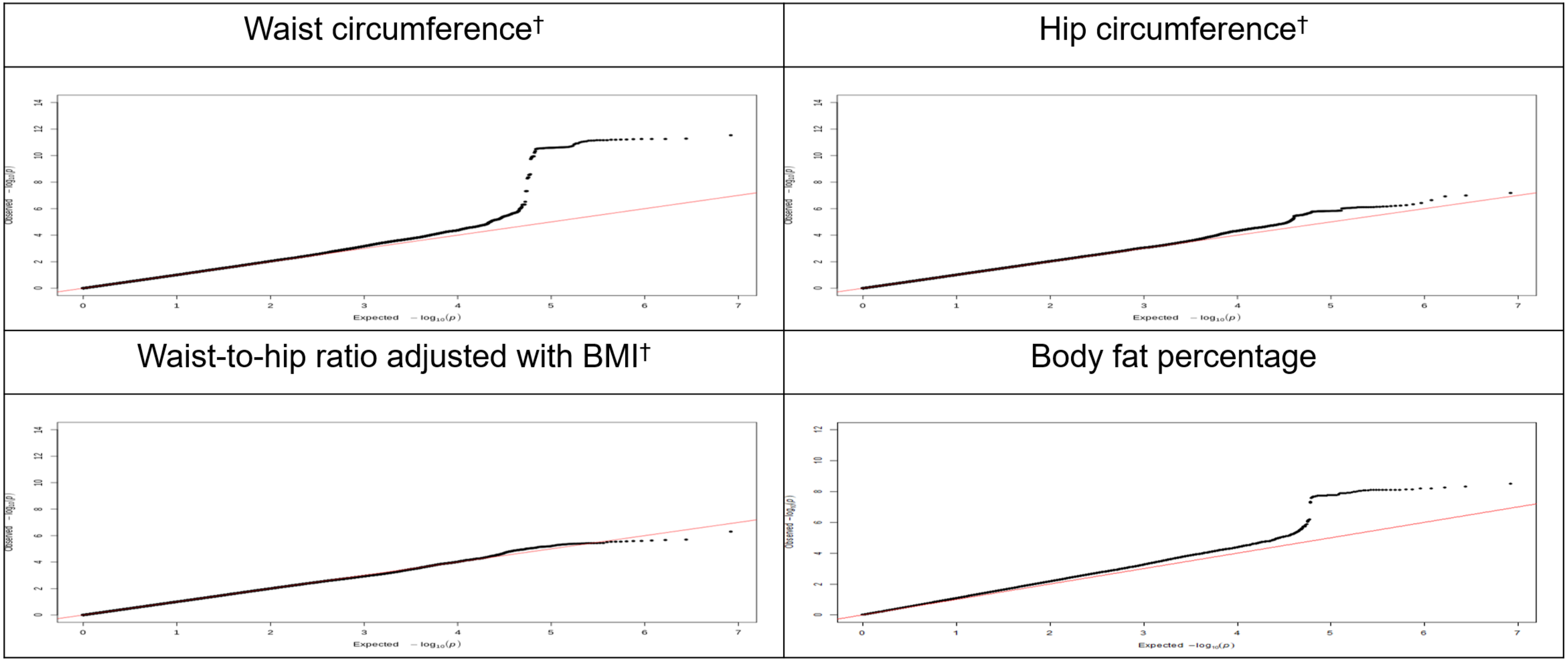

Supplementary Figure 10. Quantile-quantile plot of gene-alcohol intake frequency score interaction study in obesity-related traits. Interaction *P*-values between genetic variant and alcohol intake frequency were computed using Plink v.1.90.

† indicates GWIS after genomic control.

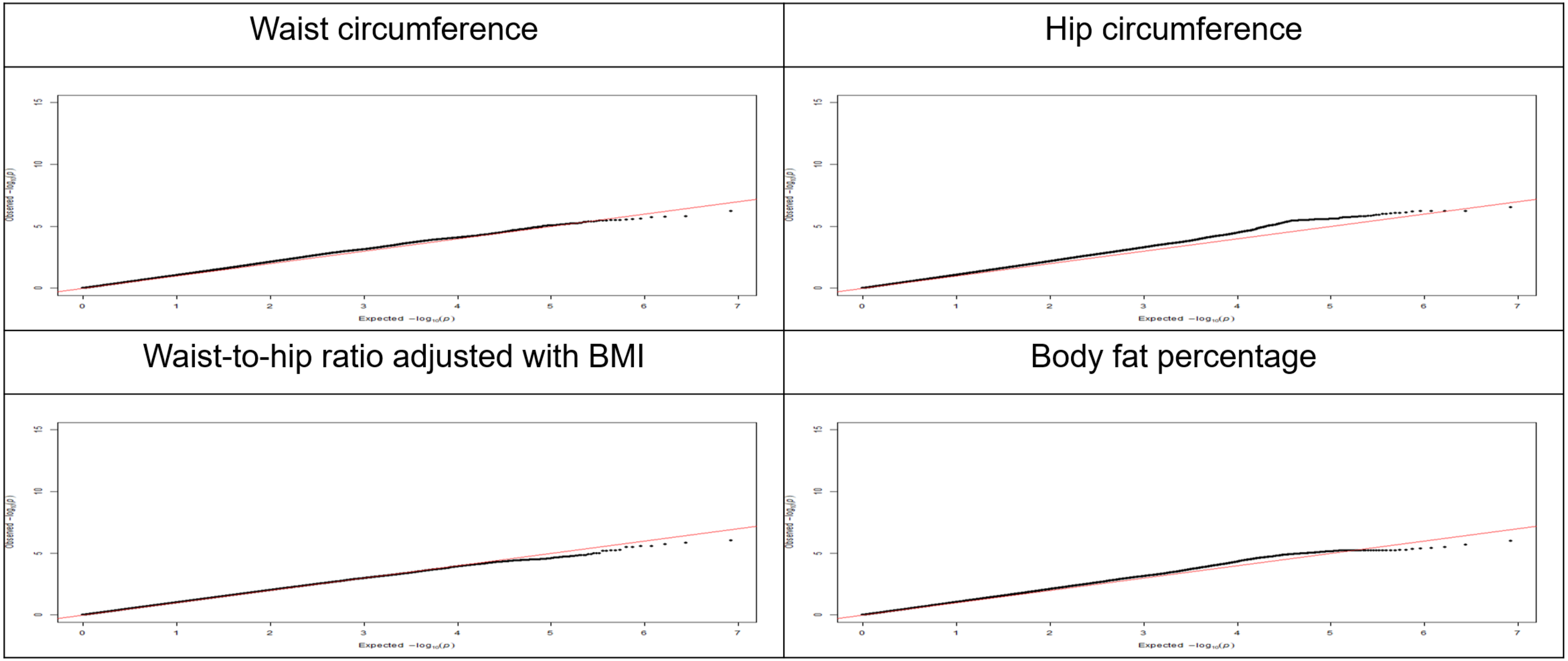

Supplementary Figure11. Quantile-quantile plot of gene-pack years of smoking score interaction study in obesity-related traits. Interaction *P*-values between genetic variant and pack years of smoking were computed using Plink v.1.90

Supplementary Table 1. Effects of lifestyle factors on body mass index (BMI).

ID	Lifestyle factor	N	*E*			*E**		
			P-value	*β*	SE	*P*-value	*β*	SE
22040	Summed MET minutes per week for all activity	268,536	< 2.00E-16	-1.54E-04	3.29E-06	< 2.00E-16	-7.60E-03	1.20E-04
884	Number of days/week of moderate physical activity 10+ minutes	315,747	< 2.00E-16	-2.14E-01	3.57E-03	< 2.00E-16	-6.73E-03	1.17E-04
1070	Time spent watching television (TV)	328,943	< 2.00E-16	6.21E-01	5.00E-03	< 2.00E-16	1.66E-02	1.28E-04
1080	Time spent using computer	328,824	< 2.00E-16	2.32E-01	6.25E-03	< 2.00E-16	3.95E-03	1.39E-04
20116	Smoking status	330,138	< 2.00E-16	2.05E-01	1.24E-02	< 2.00E-16	-6.61E-03	4.15E-04
20161	Pack years of smoking	279,758	< 2.00E-16	3.31E-02	5.77E-04	< 2.00E-16	9.08E-03	1.74E-04
20117	Alcohol intake status	330,995	< 2.00E-16	-5.25E-01	2.15E-02	< 2.00E-16	-6.90E-03	3.25E-04
1558	Alcohol intake frequency	331,049	< 2.00E-16	4.67E-01	5.59E-03	< 2.00E-16	6.70E-03	8.44E-05
20127	Neuroticism score	269,392	< 2.00E-16	3.41E-02	2.82E-03	< 2.00E-16	1.09E-03	1.24E-04
1960	Fed-up feelings	324,753	< 2.00E-16	9.70E-01	1.70E-02	< 2.00E-16	1.44E-02	2.56E-04
1160	Sleep duration	329,523	< 2.00E-16	-1.03E-01	7.60E-03	< 2.00E-16	-1.91E-03	1.33E-04
1190	Nap during day	331,140	< 2.00E-16	8.91E-01	1.40E-02	< 2.00E-16	1.31E-02	2.12E-04
738	Average total household income before tax	285,545	< 2.00E-16	-4.08E-01	7.90E-03	< 2.00E-16	-7.80E-03	1.58E-04
189	Townsend deprivation index at recruitment	330,893	< 2.00E-16	1.48E-01	2.80E-03	< 2.00E-16	5.04E-03	1.11E-04

The *E* estimates were analysed using the raw variables and the *E** estimates were analyzed using the processed variables both by the linear regression.

MET score, metabolic equivalent of task score.

Supplementary Table 2. Genomic inflation on each genome-wide interaction study.

Category	Lifestyle factor	Lambda before genomic control	LDSC intercept before genomic control	Lambda after genomic control	LDSC intercept after genomic control
Physical activity	MET score	1.13	1.08	-	-
	Moderate physical activity	1.04	1.03	-	-
Physical activity (Leisure life)	Time spent watching television (TV)	1.13	1.09	-	-
	Time spent using computer	1.08	1.09	-	-
Smoking	Smoking status	1.12	1.09	-	-
	Pack years of smoking	1.12	1.07	-	-
Alcohol	Alcohol intake stauts†	1.40	1.38	1.00	0.99
	Alcohol intake frequency†	1.21	1.17	1.00	0.96
Mental health	Neuroticism score	1.03	1.04	-	-
	Fed-up feelings	1.05	1.02	-	-
Sleep	Sleep duration†	1.18	1.17	1.00	0.98
	Nap during day	1.05	1.06	-	-
Social economic status	Average total household income before tax	1.01	1.00	-	-
	Townsend deprivation index at recruitment	1.11	1.08	-	-

† indicates the Lambda and LDSC intercept value more than 1.1.

MET score, metabolic equivalent of task score.

Supplementary Table 3. Two SNPs showing genome-wide significant G×E interactions with lifestyle factors in body mass index.

Lifestyle factor	Chromosome	Gene	SNP ID	Position[a]	Minor allele	Major allele	MAF[b] (%)	Main			G×E interaction			
								Beta	SE	*P* [c]	Beta	SE	*P* [d]	*P* [e]
Smoking status	15	*HYKK**	rs12438181	78812098	A	G	21.87	-0.000202	0.000213	0.3444	0.004690	0.000713	4.63×10^{-11}	-
Alcohol intake frequency[†]	16	*FTO**	rs11642015	53799279	T	G	42.05	0.005325	0.000179	3.24E-195	0.000829	0.000131	3.85×10^{-12}	7.24×10^{-10}

[a] Chromosomal position is based on the 1000 Genomes Project's haplotype phase 1 in NCBI build 37 (hg19).

[b] MAF is minor allele frequency calculated from subjects in this study.

[c] The *P* for the effect of association on body mass index was assessed using Plink v.1.90 with adjustment for age, sex, genotyping array, and PC1~10.

[d] The *P* for the effect of interaction between genotype and lifestyle factor on BMI was assessed using Plink v.1.9.0 with adjustment for age, sex, genotyping array, and PC1~10.

e The *P* after genomic control.

* indicates the genes within which the G×E interaction SNP is located.

† indicates the GWIS after genomic control.

MAF, minor allele frequency; SE, standard error.

Supplementary Table 4. Interactions between individual SNPs and each lifestyle factor ($P < 5.00E-06$).

Lifestyle factor	Chromosome	Nearest gene	SNP ID	Position[a]	Minor allele	Major allele	MAF[b] (%)	Beta	SE	*P*[c]	*P*[e]
MET score	1	*C1orf94*	rs967833	34687652	G	T	25.89	0.00103	0.0002197	2.77E-06	-
	1	*SNRPE*	rs118026305	203860988	A	T	12.22	-0.001378	0.0002949	2.99E-06	-
	8	*ARHGEF10**	rs74707125	1895213	T	G	9.45	0.001553	0.0003285	2.26E-06	-
	10	*ODAD2P1**	rs7075329	27600178	C	T	33.35	-0.001098	0.002030	6.41E-08	-
	10	*MIR378C*	rs7093355	132572375	T	G	11.18	0.001409	0.0003017	3.01E-06	-
	14	*DAD1**	rs28624607	23040108	A	G	14.97	-0.01234	0.0033	1.56E-06	-
	16	*FTO**	rs1421085	53800954	C	T	39.43	-0.000998	0.0001944	2.87E-07	-
	20	*MIR646HG**	rs17728377	58852966	A	C	11.11	-0.001406	0.0003033	3.58E-06	-
Moderate physical activity	2	*AC012501.2*	rs75708282	154407649	G	C	7.09	0.001785	0.0003208	2.64E-08	-
	3	*RP11-6F2.4*	rs55730982	156797208	G	T	39.91	0.0008938	0.0001693	1.31E-07	-
	9	*LOC105375970**	rs1952178	7343188	T	C	28.54	-0.0008584	0.0001847	3.38E-06	-
	9	*ASS1*	rs688943	133395013	C	G	17.67	-0.0011	0.0002172	4.09E-07	-
	16	*FTO**	rs1421085	53800954	C	T	39.43	-0.0008339	0.0001682	7.19E-07	-
	20	*MIR646HG**	rs62206729	58832180	C	T	11.78	-0.001278	0.0002582	7.38E-07	-
Time spent watching TV	3	*XYLB**	rs62239899	38438099	G	A	7.973	0.001584	0.0003285	1.43E-06	-
	4	*KCNIP4**	rs61420026	21269183	G	C	9.781	-0.001414	0.000298	2.09E-06	-
	5	*SSBP2**	rs1373966	81007502	T	C	12.33	-0.001242	0.0002692	3.94E-06	-
	6	*PRPF4B**	rs2181894	4022611	A	G	22.43	0.0009734	0.0002126	4.67E-06	-
	6	*RP1-27K12.2*	rs738472	53477038	C	T	35.2	0.0008516	0.0001863	4.85E-06	-
	6	*HIVEP2**	rs13195550	143159284	T	C	32.14	-0.0009099	0.0001915	2.02E-06	-
	7	*HIP1**	rs1182890	75271709	C	T	11.94	0.001401	0.0002728	2.79E-07	-
	7	*HGF*	rs10228618	81472280	C	T	31.13	0.0009085	0.0001928	2.45E-06	-
	7	*FAM131B**	rs56242860	143068887	T	C	23.73	-0.00101	0.000209	1.34E-06	-
	9	*TRPM3**	rs3812535	73222151	C	T	12.84	0.00131	0.0002669	9.22E-07	-
	10	*FRMD4A**	rs2614152	14131349	T	C	42.03	-0.0008436	0.0001828	3.94E-06	-
	14	*CATSPERB**	rs2750475	92130048	A	G	48.56	-0.0008382	0.0001771	2.23E-06	-
	16	*GLIS2*	rs4786481	4353432	T	C	24.05	0.001014	0.0002109	1.53E-06	-
	16	*FTO**	rs7201850	53821862	T	C	41.28	0.0009537	0.0001799	1.15E-07	-
	19	*WTIP*	rs73032050	35013701	A	G	5.801	0.001767	0.0003793	3.17E-06	-
	1	*LINC01137*	rs35010804	37874498	G	C	9.284	0.001532	0.0003347	4.72E-06	-

	1	*H3F3A*	rs6659731	226233187	G	A	5.194	-0.002432	0.000441	3.52E-08	-
	2	*EML6*	rs79747576	54934844	A	C	5.35	-0.002064	0.0004305	1.64E-06	-
	3	*FHIT**	rs17325940	60071129	C	T	12.41	0.001418	0.0002964	1.72E-06	-
	4	*LOC105377614*	rs6553310	190295623	C	A	5.954	0.002083	0.0004106	3.92E-07	-
	7	*PKD1L1**	rs6955251	47973034	A	G	21.48	-0.001167	0.0002359	7.52E-07	-
Time spent using computer	7	*PTN**	rs75193610	136996938	A	T	4.99	-0.002043	0.0004468	4.81E-06	-
	9	*LOC105376205**	rs80075325	110366264	T	C	16.52	-0.001237	0.0002617	2.27E-06	-
	12	*CLEC12B**	rs9300222	10159541	C	T	36.29	-0.000922	0.0002019	4.94E-06	-
	12	*PPFIBP1**	rs2288225	27809900	C	T	7.193	-0.001809	0.000377	1.59E-06	-
	13	*LOC105370228**	rs73542920	60927302	G	A	10.57	0.001451	0.0003159	4.36E-06	-
	17	*MED24**	rs3935280	38189055	A	G	38.81	-0.000925	0.0001994	3.51E-06	-
	18	*LINC01254*	rs1529533	10405984	A	G	38.94	-0.001008	0.0001984	3.79E-07	-
	2	*VIT**	rs112233816	36944668	T	C	16.36	0.00369	0.0007934	3.32E-06	-
	2	*SNORD112*	rs72838848	84120300	A	G	11.15	-0.004415	0.0009265	1.89E-06	-
	3	*VGLL3*	rs17023854	87104899	C	T	35.85	0.002839	0.0006131	3.64E-06	-
	6	*RP11-686D16.1*	rs13191185	19366348	A	G	6.767	-0.00557	0.001193	3.04E-06	-
	7	*CCM2**	rs73107935	45075512	A	G	13.9	-0.003849	0.0008393	4.51E-06	-
Smoking status	8	*RP11-127H5.1*	rs62525303	106281276	G	A	29.45	0.003556	0.0006478	4.03E-08	-
	11	*RP11-136I14.5*	rs11215622	115470537	T	C	28.29	0.003079	0.0006535	2.46E-06	-
	13	*MYO16**	rs9521081	109534382	C	G	36.05	-0.002901	0.0006099	1.98E-06	-
	14	*LOC105370643**	rs34210449	96054603	A	G	12.58	0.004252	0.000885	1.56E-06	-
	15	*HYKK**	rs12438181	78812098	A	G	21.87	0.00469	0.0007125	4.63E-11	-
	17	*CA10**	rs2191405	50129900	A	T	11.6	-0.004339	0.000911	1.90E-06	-
	1	*RP13-279N23.2*	rs72651851	19186735	T	G	8.672	0.00213	0.0004287	6.72E-07	-
	1	*RNF220**	rs2236219	45101478	C	T	38.63	0.001194	0.0002497	1.72E-06	-
	1	*RP11-309H21.3*	rs71637266	191111903	T	C	16.87	0.00168	0.0003263	2.64E-07	-
	2	*LOC105373611**	rs72847808	129197248	C	G	18.11	-0.001452	0.0003133	3.60E-06	-
	3	*DCBLD2**	rs2439237	98572853	T	C	47.5	0.001111	0.000242	4.45E-06	-
	4	*STK32B**	rs1447287	5202206	C	G	46.94	0.001173	0.0002451	1.71E-06	-
	7	*POU6F2**	rs73126493	39300905	T	C	36.58	-0.001187	0.0002553	3.32E-06	-
Pack years of smoking	9	*RP11-281A20.*	rs4837532	120964337	T	G	34.87	-0.00124	0.0002556	1.22E-06	-
	9	*FAM163B**	rs113927489	136467207	G	C	11.21	-0.002043	0.0003781	6.60E-08	-
	11	*SOX6**	rs10832651	16634967	A	C	20.62	0.001452	0.000301	1.41E-06	-
	14	*NRXN3**	rs8008994	79643268	T	G	27.75	-0.001259	0.0002725	3.82E-06	-
	15	*CHRNA3**	rs4243084	78911672	C	G	33.86	-0.00142	0.0002536	2.15E-08	-
	16	*CTD-2535I10.1*	rs28495409	7893107	A	G	7.607	-0.002233	0.0004551	9.24E-07	-
	16	*COG8**	rs72795276	69366986	A	G	6.487	-0.002322	0.0004897	2.13E-06	-
	17	*ASIC2**	rs2228990	32483237	G	A	19.44	0.001564	0.0003039	2.64E-07	-

	17	*LINC02210-CRHR1**	rs35116560	43804186	C	T	41.61	-0.001171	0.0002497	2.75E-06	-
	17	*TIMP2*	rs4789931	76926392	T	C	32.78	-0.001194	0.0002578	3.65E-06	-
Alcohol intake status†	7	*SKAP2**	rs2893256	26753368	A	G	36.94	0.002611	0.000477	4.40E-08	3.81E-06
	7	*RELN**	rs12705172	103600984	T	G	42.06	0.002586	0.0004712	4.05E-08	3.59E-06
	15	*FAM98B*	rs4479187	38711486	T	C	40.43	-0.002872	0.0004688	8.97E-10	2.31E-07
Alcohol intake frequency†	2	*SCHLAP1**	rs11677686	181561742	T	C	36.85	-0.000645	0.0001228	1.49E-07	1.80E-06
	4	*ZFP42*	rs59266038	188770462	G	T	8.296	0.0132	0.002637	5.59E-07	4.93E-06
	5	*CTNND2**	rs2642712	11229591	T	C	37.51	-0.007321	0.001522	1.50E-06	2.12E-06
	5	*RP11-152K4.2*	rs10044831	31162365	G	A	9.407	0.0117	0.002485	2.52E-06	1.73E-06
	5	*C5orf60**	rs6891180	179072417	T	C	43.53	-0.007541	0.001464	2.47E-07	1.84E-06
	7	*EXOC4**	rs13240997	133137753	A	C	30.5	0.007429	0.001571	2.25E-06	3.12E-06
	16	*FTO**	rs11642015	53802494	T	C	39.45	0.0008286	0.0001194	3.85E-12	7.24E-10
Neuroticism score	2	*HS6ST1**	rs8179791	129056917	C	T	49.22	0.0009478	0.0002044	3.53E-06	-
	5	*MFAP3*	rs35987822	153468552	C	G	5.26	-0.002363	0.000456	2.20E-07	-
	6	*PXDC1*	rs62392431	3784555	A	G	5.07	0.002156	0.0004644	3.46E-06	-
	7	*AUTS2**	rs7783682	70198964	C	G	38.83	0.0009725	0.0002125	4.76E-06	-
	17	*CCDC92B**	rs11654129	2646490	A	G	37.93	0.00101	0.000211	1.72E-06	-
Fed-up feelings	4	*ENPEP*	rs244022	111250939	A	C	30.97	0.00181	0.0003929	4.09E-06	-
	6	*LINC02542**	rs2210978	82754922	A	G	7.696	0.003266	0.0006817	1.66E-06	-
	7	*GTF2IRD1P1**	rs4718413	66292459	T	G	16.99	0.002304	0.0004769	1.36E-06	-
	8	*DLGAP2**	rs112307130	962483	A	G	32.95	0.001841	0.0003914	2.57E-06	-
	11	*DISC1FP1**	rs472190	90034660	G	A	27.14	-0.001902	0.000408	3.14E-06	-
	14	*KCNK10**	rs8009725	88661798	A	G	45.63	0.001729	0.0003618	1.78E-06	-
	20	*SDCBP2**	rs2146662	1297516	A	G	28.2	0.002057	0.0004028	3.27E-07	-
Sleep duration†	5	*LOC105379035**	rs255584	73342958	A	G	33.85	-0.000991	0.0001986	6.01E-07	4.54E-06
	7	*DOCK4*	rs34587256	111337540	G	T	9.717	-0.001647	0.0003217	3.05E-07	2.55E-06
	13	*EPSTI1**	rs74406744	43477211	A	C	7.665	0.001777	0.0003529	4.74E-07	3.71E-06
	15	*PWRN4**	rs328646	24257279	A	G	14.25	-0.001496	0.0002689	2.64E-08	3.20E-07
Nap during day	1	*GLUL*	rs3001278	182325045	C	T	14.27	-0.001991	0.0004176	1.86E-06	-
	1	*RP11-358H9.1*	rs61824117	222648295	T	C	45.57	0.00147	0.0003002	9.78E-07	-
	2	*LOC105369165**	rs74606066	53209306	T	C	16.93	0.001787	0.0003913	4.97E-06	-
	2	*AC005042.5*	rs1913900	159617573	T	C	16.31	0.00181	0.0003963	4.92E-06	-
	4	*LOC107986178**	rs17503535	11652332	T	C	5.238	-0.003076	0.000669	4.27E-06	-
	5	*PCDHA9**	rs113766408	140292797	G	T	11.34	-0.002238	0.0004639	1.40E-06	-
	6	*BLOC1S5**	rs12663094	8018417	A	G	17.09	0.001827	0.0003886	2.60E-06	-
	11	*NELL1**	rs78583883	20692597	A	G	11.28	0.002131	0.0004643	4.44E-06	-
	14	*LINC01550**	rs72702226	98414097	T	G	8.223	0.002465	0.0005341	3.94E-06	-
	15	*MEIS2**	rs71468837	37351922	T	G	22.25	0.001624	0.0003518	3.93E-06	-

	15	*RP11-718O11.1*	rs12441402	46326327	C	G	40.94	0.001368	0.0002991	4.81E-06	-
	16	*FTO**	rs8047395	53798523	A	G	49.96	0.001524	0.000294	2.16E-07	-
	18	*LINC01443**	rs4890740	14967230	C	G	34.99	0.001427	0.0003112	4.52E-06	-
	20	*C20orf27**	rs75077214	3747759	G	T	9.851	-0.002294	0.000497	3.91E-06	-
Average total household income before tax	2	*CERS6**	rs17249307	169510400	G	C	18.28	-0.000947	0.0002064	4.51E-06	-
	4	*TAPT1**	rs894639	16226298	G	A	39.16	-0.000781	0.0001614	1.31E-06	-
	4	*FAT4**	rs6822253	126409715	C	T	40.28	0.0007353	0.0001608	4.81E-06	-
	7	*RP11-153N17.1*	rs59430777	52215260	C	T	29.79	-0.000827	0.0001736	1.92E-06	-
	7	*AC004014.3*	rs1513923	108624503	G	A	20.69	-0.000895	0.0001947	4.23E-06	-
	20	*RP11-151E14.1*	rs6071415	59477515	G	C	25.89	-0.000845	0.0001831	3.94E-06	-
TDI	1	*LGR6**	rs3010075	202226701	T	C	33.85	-0.000893	0.0001847	1.33E-06	-
	1	*LOC105372912**	rs35870652	213961934	C	T	6.348	-0.001659	0.0003621	4.59E-06	-
	3	*AC027119.1*	rs1347421	5891563	T	A	27.88	0.0009151	0.0001964	3.16E-06	-
	5	*PDE4D**	rs13162864	59207614	C	A	12.78	0.001196	0.0002617	4.86E-06	-
	7	*ASL**	rs6967430	65548725	A	G	5.557	0.001817	0.0003815	1.91E-06	-
	8	*LOC107986945**	rs1876380	59123769	T	C	11.12	0.001374	0.0002789	8.39E-07	-
	9	*TEK**	rs578327	27193847	G	A	25.32	0.0009901	0.0002017	9.19E-07	-
	10	*RP11-543F8.2*	rs4747797	8557486	G	C	13.17	-0.001301	0.0002588	4.95E-07	-
	11	*RP11-379J13.2*	rs2851655	97731658	A	T	18.41	-0.00106	0.0002272	3.07E-06	-
	12	*AC023050.1*	rs1259773	32077340	G	A	27.4	0.0009145	0.0001959	3.03E-06	-
	15	*LINC00924**	rs2052653	96002970	A	T	15.41	0.001136	0.000245	3.55E-06	-
	16	*HS3ST4**	rs9937575	25954603	G	A	16.66	0.001117	0.0002365	2.32E-06	-
	19	*ZBTB7A**	rs188955288	4067314	T	C	18.58	-0.001132	0.0002274	6.50E-07	-
	19	*CNOT3*	rs111714632	54639342	T	A	38.05	-0.000845	0.0001825	3.65E-06	-
	22	*C22orf34*	rs10212061	49579609	A	G	13.28	-0.001182	0.0002582	4.68E-06	-

[a] Chromosomal position is based on the 1000 Genomes Project's haplotype phase 1 in NCBI build 37 (hg19).

[b] MAF is minor allele frequency calculated from subjects in this study.

[c] The *P*-value for the effect of interaction between genotype and lifestyle factor on BMI was assessed by using Plink v.1.9.0 with adjustment for age, sex, genotyping array and PC1~10.

[e] The P after genomic control.

* indicates the genes within which the GxE interaction SNP locates.

† indicates the GWIS after genomic control.

MET score, Metabolic equivalent of task score; TDI, Townsend deprivation index at recruitment; MAF, minor allele frequency; SE, standard error.

Supplementary Table 5. Functional annotation of two SNPs with genome-wide significant level for multiple testing (*P* -value < 3.57E-09 calculated by 5.00E-08 / 14) on BMI.

Lifestyle factor	Chromosome	Nearest gene	SNP ID	dbSNP functional annotation	RegulomeDB score[a]	RegoulomeDB rank[b]
Smoking status	15	*HYKK**	rs12438181	intronic	0.13	5
Alcohol intake frequency[†]	16	*FTO**	rs11642015	intronic	0.19	6

[a] The RegulomeDB score represents a model integrating functional genomics features along with continuous values such as ChIP-seq signal, DNase-seq signal, information content change, and DeepSEA scores.

[b] RegulomeDB rank refers to the following available datatypes for a single coordinate:

5 – TF binding or DNase peak, 6 – Motif hit.

* indicates the genes within which the G×E interaction SNP locates.

† indicates the GWIS after genomic control.

BMI, body mass index.

Supplementary Table 6. Previously reported association with obesity or obesity-related tratis based on genetic variants within the 1 Mb flanking regions of G×E interaction SNPs.

Lifestyle factor	Chromosome	Nearest gene	SNP ID	BP	Reported SNP ID	Reported SNP BP	Reported traits	Reported *P* -value	Authors	PubMed ID	Title
Smoking status	15	*HYKK**	rs12438181	78812098	rs2870111	79403585	Body mass index	4.00E-12	Pulit SL	30239722	Meta-analysis of genome-wide association studies for body fat distribution in 694,649 individuals of European ancestry.
Alcohol intake frequency†	16	*FTO**	rs11642015	53799279	rs11642015	53768582	Body mass index	2.00E-81	Pulit SL	30239722	Meta-analysis of genome-wide association studies for body fat distribution in 694,649 individuals of European ancestry.

* indicates the genes within which the G×E interaction SNP locates

† indicates the GWIS after genomic control.

Supplementary Table 7. Previously reported association of G×E interaction two SNPs with genome-wide significant level for multiple testing (P < 3.57E-09 calculated by 5.00E-08 / 14) on BMI.

Lifestyle factor	Chromosome	Nearest gene	SNP ID	BP	Reported traits	Reported *P* -value	Authors	PubMed ID	Title
Smoking status	15	*HYKK**	rs12438181	78812098	Forced expiratory volume, response to bronchodilator	1.00E-06	Lutz SM	26634245	A genome-wide association study identifies risk loci for spirometric measures among smokers of European and African ancestry.
					Cigarettes per day measurement	5.00E-10	Liu M	30643251	Association studies of up to 1.2 million individuals yield new insights into the genetic etiology of tobacco and alcohol use.
Alcohol intake frequency[†]	16	*FTO**	rs11642015	53799279	Ease of getting up in the morning	6.00E-11	Jansen PR	30804565	Genome-wide analysis of insomnia in 1,331,010 individuals identifies new risk loci and functional pathways.
					Type 2 diabetes	1.00E-35	Ishigaki K	32514122	Large-scale genome-wide association study in a Japanese population identifies novel susceptibility loci across different diseases.
					Hand grip strength	8.00E-49	Tikkanen E	29691431	Biological Insights Into Muscular Strength: Genetic Findings in the UK Biobank.
					Diastolic blood pressure x smoking status (current vs non-current) interaction (2df test)	3.00E-11	Sung YJ	29455858	A Large-Scale Multi-ancestry Genome-wide Study Accounting for Smoking Behavior Identifies Multiple Significant Loci for Blood Pressure.
					Diastolic blood pressure x smoking status (ever vs never) interaction (2df test)	2.00E-10	Sung YJ	29455858	A Large-Scale Multi-ancestry Genome-wide Study Accounting for Smoking Behavior Identifies Multiple Significant Loci for Blood Pressure.
					Systolic blood pressure x smoking status (ever vs never) interaction (2df test)	4.00E-20	Sung YJ	29455858	A Large-Scale Multi-ancestry Genome-wide Study Accounting for Smoking Behavior Identifies Multiple Significant Loci for Blood Pressure.
					Diastolic blood pressure x smoking status (current vs non-current) interaction (2df test)	3.00E-11	Sung YJ	29455858	A Large-Scale Multi-ancestry Genome-wide Study Accounting for Smoking Behavior Identifies Multiple Significant Loci for Blood Pressure.
					Systolic blood pressure x smoking status (current vs non-current) interaction (2df test)	3.00E-11	Sung YJ	29455858	A Large-Scale Multi-ancestry Genome-wide Study Accounting for Smoking Behavior Identifies Multiple Significant Loci for Blood Pressure.
					Body mass index	9.00E-290	Sakaue S	34594039	A cross-population atlas of genetic associations for 220 human phenotypes.
					Blood urea nitrogen levels	6.00E-16	Sakaue S	34594039	A cross-population atlas of genetic associations for 220 human phenotypes.
					Mean arterial pressure	1.00E-09	Takeuchi F	30487518	Interethnic analyses of blood pressure loci in populations of East Asian and European descent.
					Urinary sodium excretion	7.00E-23	Pazoki R	31409800	GWAS for urinary sodium and potassium excretion highlights pathways shared with cardiovascular traits.
					Systolic blood pressure	2.00E-12	Takeuchi F	30487518	Interethnic analyses of blood pressure loci in populations of East Asian and European descent.
					Diastolic blood pressure	3.00E-06	Takeuchi F	30487518	Interethnic analyses of blood pressure loci in populations of East Asian and European descent.
					Pulse pressure	3.00E-10	Takeuchi F	30487518	Interethnic analyses of blood pressure loci in populations of East Asian and European descent.
					Body mass index variance	2.00E-73	Wang H	31453325	Genotype-by-environment interactions inferred from genetic effects on phenotypic variability in the UK Biobank.
					Menarche (age at onset)	1.00E-57	Kichaev G	30595370	Leveraging Polygenic Functional Enrichment to Improve GWAS Power.

* indicates the genes within which the G×E interaction SNP locates

† indicates the GWIS after genomic control.

Supplementary Table 8. The eQTL information of G×E interaction two SNPs with genome-wide significant level for multiple testing (P < 3.57E-09 calculated by 5.00E-08 / 14) on BMI.

Chromosome	Position	Sentinel SNP	Locus	Tested allele	Direction	Gene	eQTL *P*-value	eQTL effect size	Tissue
					+	*PSMA4*	2.80E-40	0.26	Muscle - Skeletal
					-	*CHRNA5*	7.60E-23	-0.58	Muscle - Skeletal
					-	*RP11-650L12.4*	2.40E-21	-0.45	Testis
					-	*CHRNA3*	3.50E-14	-0.43	Muscle - Skeletal
					-	*PSMA4*	3.60E-13	-0.12	Whole Blood
					+	*IREB2*	3.90E-12	0.18	Skin - Not Sun Exposed (Suprapubic)
					-	*CHRNA5*	3.90E-10	-0.43	Nerve - Tibial
					-	*CHRNA5*	9.10E-10	-0.29	Cells - Cultured fibroblasts
					+	*IREB2*	7.30E-09	0.15	Skin - Sun Exposed (Lower leg)
					-	*CHRNA5*	8.10E-09	-0.41	Heart - Left Ventricle
					-	*RP11-650L12.2*	7.30E-08	-0.26	Cells - Cultured fibroblasts
					-	*CHRNA5*	9.30E-08	-0.36	Adipose - Visceral (Omentum)
					-	*CHRNA3*	1.20E-07	-0.39	Nerve - Tibial
					+	*PSMA4*	2.40E-07	0.12	Artery - Tibial
					-	*CHRNA5*	2.90E-07	-0.59	Brain - Frontal Cortex (BA9)
					-	*CHRNA5*	4.20E-07	-0.34	Adipose - Subcutaneous
					+	*PSMA4*	7.30E-07	0.18	Heart - Left Ventricle
15	78812098	rs12438181	*HYKK*	G	-	*RP11-650L12.4*	1.40E-06	-0.33	Thyroid
					-	*CHRNA5*	1.60E-06	-0.62	Brain - Hypothalamus
					-	*CHRNA3*	1.90E-06	-0.45	Brain - Caudate (basal ganglia)
					-	*CHRNA3*	2.10E-06	-0.47	Brain - Nucleus accumbens (basal ganglia)
					+	*PSMA4*	3.20E-06	0.17	Heart - Atrial Appendage
					-	*RP11-650L12.4*	5.10E-06	-0.4	Colon - Transverse
					-	*ADAMTS7P3*	5.50E-06	-0.31	Cells - Cultured fibroblasts
					-	*CHRNA5*	6.50E-06	-0.52	Brain - Nucleus accumbens (basal ganglia)
					-	*CHRNA5*	6.70E-06	-0.26	Artery - Tibial
					-	*CHRNA3*	7.30E-06	-0.34	Testis
					-	*CHRNA5*	7.60E-06	-0.58	Brain - Hippocampus
					-	*CHRNA5*	8.70E-06	-0.44	Artery - Coronary
					+	*IREB2*	1.20E-05	0.12	Thyroid
					+	*RP11-650L12.2*	1.30E-05	0.23	Colon - Sigmoid
					+	*IREB2*	1.30E-05	0.098	Lung
					+	*RP11-650L12.2*	1.40E-05	0.22	Colon - Transverse
					+	*HYKK*	1.70E-05	0.4	Brain - Caudate (basal ganglia)
					-	*CHRNA5*	2.70E-05	-0.37	Heart - Atrial Appendage

					-	*RP11-650L12.2*	7.60E-05	-0.27	Adipose - Subcutaneous
					-	*CHRNA5*	8.20E-05	-0.22	Esophagus - Mucosa
					+	*HYKK*	1.00E-04	0.13	Muscle - Skeletal
					-	*RP11-650L12.4*	1.20E-04	-0.26	Adipose - Subcutaneous
					+	*PSMA4*	1.30E-04	0.1	Skin - Not Sun Exposed (Suprapubic)
					+	*IREB2*	1.70E-04	0.093	Adipose - Subcutaneous
16	53802494	rs11642015	*FTO*	C	+	*FTO*	6.30E-08	0.14	Muscle - Skeletal
					+	*IRX3*	1.10E-05	0.36	Pancreas

† indicates the GWIS after genomic control.
BMI, body mass index.

Supplementary Table 9. Basic characteristics of participants for waist circumference.

Group	Quartile 1 group	Quartile 2 group	Quartile 3 group	Quartile 4 group
Female threshold	WC ≤ 75	75 < WC ≤ 83	83 < WC ≤ 92	92 < WC
Male threshold	WC ≤ 89	89 < WC ≤ 96	96 < WC ≤ 103	103 < WC
Number of participants	84,098	88,233	78,402	80,485
Males (%)	46.22	45.92	45.25	47.55
Age at assessment center (years) (mean, SD)	55.23 ± 8.20	56.68 ± 7.99	57.57 ± 7.77	57.87 ± 7.57
BMI (kg/m^2) (mean, SD)	23.01 ± 2.23	25.68 ± 2.16	28.11 ± 2.43	33.13 ± 4.47
Waist circumference (cm) (mean, SD)	76.73 ± 7.81	85.71 ± 7.12	93.14 ± 6.45	106.73 ± 9.54
MET score				
MET score mean (SD)	3032.61 ± 2846.03	2750.21 ± 2694.55	2589.64 ± 2664.06	2238.58 ± 2544.24
Alcohol intake frequency				
Category mean (SD)	2.75 ± 1.45	2.71 ± 1.43	2.82 ± 1.47	3.11 ± 1.54
Pack years of smoking				
Pack years of smoking mean (SD)	5.55 ± 12.47	6.98 ± 13.79	8.85 ± 15.92	12.20 ± 19.70

Each quartile group is a combination of men and women equivalent to the corresponding quartile.

BMI, Body mass index; MET score, metabolic equivalent of task score; SD, standard deviation; WC, waist circumference.

Supplementary Table 10. Basic characteristics of participants for hip circumference.

Group	Quartile 1 group	Quartile 2 group	Quartile 3 group	Quartile 4 group
Female threshold	HC ≤ 96	96 < HC ≤ 102	102 < HC ≤ 108	108 < HC
Male threshold	HC ≤ 99	99 < HC ≤ 103	103 < HC ≤ 107	107 < HC
Number of participants	90,361	86,221	71,373	82,516
Males (%)	50.02	43.29	45.57	46.16
Age at assessment center (years) (mean, SD)	56.56 ± 8.06	56.82 ± 8.00	57.00 ± 7.92	56.88 ± 7.84
BMI (kg/m^2) (mean, SD)	23.40 ± 2.54	25.57 ± 2.48	27.99 ± 2.51	32.93 ± 4.54
Hip circumference (cm) (mean, SD)	93.97 ± 3.64	99.84 ± 2.07	105.31 ± 1.45	115.39 ± 7.71
MET score				
MET score mean (SD)	3019.38 ± 2893.65	2741.46 ± 2682.16	2580.64 ± 2632.06	2259.03 ± 2521.87
Alcohol intake frequency				
Category mean (SD)	2.73 ± 1.47	2.75 ± 1.44	2.82 ± 1.45	3.08 ± 1.51
Pack years of smoking				
Pack years of smoking mean (SD)	7.73 ± 15.13	7.40 ± 14.71	8.17 ± 1.46	10.08 ± 17.82

Each quartile group is a combination of men and women equivalent to the corresponding quartile.

BMI, Body mass index; MET score, metabolic equivalent of task score; SD, standard deviation; HC, hip circumference.

Supplementary Table 11. Basic characteristics of participants for body fat percentage.

Group	Quartile 1 group	Quartile 2 group	Quartile 3 group	Quartile 4 group
Female threshold	BFP ≤ 32	32 < BFP ≤ 36.7	36.7 < BFP ≤ 41.3	41.3 < BFP
Male threshold	BFP ≤ 21.5	21.5 < BFP ≤ 25.4	25.4 < BFP ≤ 29.1	29.1 < BFP
Number of participants	81,872	82,305	81,396	80,727
Males (%)	46.05	46.92	45.88	47.55
Age at assessment center (years) (mean, SD)	54.68 ± 8.20	56.55 ± 7.98	57.65 ± 7.69	58.32 ± 7.45
BMI (kg/m^2) (mean, SD)	23.04 ± 2.31	25.66 ± 2.23	28.00 ± 2.48	32.92 ± 4.52
Body fat percentage (mean, SD)	23.16 ± 5.97	29.39 ± 5.58	33.58 ± 5.98	39.38 ± 6.94
MET score				
MET score mean (SD)	2525.29 ± 2626.62	2786.21 ± 2733.61	2563.14 ± 2621.84	2193.53 ± 2468.00
Alcohol intake frequency				
Category mean (SD)	2.71 ± 1.45	2.72 ± 1.43	2.83 ± 1.47	3.11 ± 1.53
Pack years of smoking				
Pack years of smoking mean (SD)	5.79 ± 12.57	7.00 ± 13.92	8.75 ± 15.80	11.70 ± 19.41

Each quartile group is a combination of men and women equivalent to the corresponding quartile.

BMI, Body mass index; MET score, metabolic equivalent of task score; SD, standard deviation; BFP, body fat percentage.

Supplementary Table 12. Basic characteristics of participants for waist-to-hip ratio adjusted for BMI (WHRadjBMI).

Group	Quartile 1 group	Quartile 2 group	Quartile 3 group	Quartile 4 group
Female threshold	WHRadjBMI ≤ 0.77	0.77 < WHRadjBMI ≤ 0.81	0.81 < WHRadjBMI ≤ 0.86	0.86 < WHRadjBMI
Male threshold	WHRadjBMI ≤ 0.89	0.89 < WHRadjBMI ≤ 0.93	0.93 < WHRadjBMI ≤ 0.98	0.98 < WHRadjBMI
Number of participants	84,580	76,556	88,338	81,723
Males (%)	42.87	48.19	49.61	44.24
Age at assessment center (years) (mean, SD)	54.82 ± 8.22	56.33 ± 8.02	57.55 ± 7.71	58.53 ± 7.38
BMI (kg/m^2) (mean, SD)	24.37 ± 3.39	26.33 ± 3.82	28.14 ± 4.25	30.69 ± 4.91
WHRadjBMI (mean, SD)	0.79 ± 0.065	0.85 ± 0.061	0.89 ± 0.061	0.96 ± 0.069
MET score				
MET score mean (SD)	2895.63 ± 2754.62	2741.68 ± 2711.61	2624.11 ± 2713.37	2387.25 ± 2623.09
Alcohol intake frequency				
Category mean (SD)	2.79 ± 1.44	2.75 ± 1.44	2.81 ± 1.47	3.02 ± 1.55
Pack years of smoking				
Pack years of smoking mean (SD)	4.66 ± 10.99	6.74 ± 13.41	9.25 ± 16.40	12.65 ± 19.92

Each quartile group is a combination of men and women equivalent to the corresponding quartile.

BMI, Body mass index; MET score, metabolic equivalent of task score; SD, standard deviation; WHRadjBMI, waist to hip ratio adjusted for body mass index.

Supplementary Table 13. Effects of lifestyle factors on obesity-related traits.

Phenotype	Lifestyle factor ID	Lifestyle factor	N	*E*			*E**		
				P	*β*	SE	*P*	*β*	SE
Waist circumference	22040	MET score	268,484	< 2E-16	-5.15E-04	8.28E-06	< 2E-16	-1.19E-01	1.48E-03
	20161	Pack years of smoking	279,704	< 2E-16	1.13E-01	1.44E-03	< 2E-16	1.54E-01	2.12E-03
	1558	Alcohol intake frequency	330,986	< 2E-16	9.36E-01	1.41E-02	< 2E-16	6.60E-02	1.04E-03
Hip circumference	22040	MET score	268,487	< 2E-16	-3.65E-04	6.32E-06	< 2E-16	-1.42E-01	1.89E-03
	20161	Pack years of smoking	279,707	< 2E-16	3.63E-02	1.12E-03	< 2E-16	6.21E-02	2.43E-03
	1558	Alcohol intake frequency	330,996	< 2E-16	6.97E-01	1.08E-02	< 2E-16	6.69E-02	1.19E-03
WHRadjBMI	22040	MET score	268,465	< 2E-16	-1.90E-06	4.70E-08	< 2E-16	-7.10E-02	1.94E-03
	20161	Pack years of smoking	279,684	< 2E-16	7.70E-04	8.06E-06	< 2E-16	1.69E-01	1.81E-03
	1558	Alcohol intake frequency	330,965	< 2E-16	3.14E-03	7.92E-05	< 2E-16	7.86E-02	1.19E-03
Body fat percentage	22040	MET score	264,667	< 2E-16	-3.13E-04	4.45E-06	< 2E-16	-1.02E-01	1.12E-03
	20161	Pack years of smoking	275,884	< 2E-16	4.81E-02	7.75E-04	< 2E-16	8.33E-02	1.42E-03
	1558	Alcohol intake frequency	326,077	< 2E-16	4.67E+00	7.55E-03	< 2E-16	3.83E-02	6.96E-04

The *E* estimates were analysed using the raw variables and the *E** estimates were analyzed using the processed variables both by the linear regression.
MET score, Metabolic equivalent of task score; SE, standard error; WHRadjBMI, waist to hip ratio adjusted for body mass index.

Supplementary Table 14. Genomic inflation on each genome-wide interaction study.

Phenotype	Lifestyle factor	Lambda before genomic control	LDSC intercept before genomic control	Lambda after genomic control	LDSC intercept after genomic control
Waist circumference	MET score	1.09	1.06	-	-
	Pack years of smoking	1.06	1.03	-	-
	Alcohol intake frequency†	1.19	1.16	1.00	0.97
Hip circumference	MET score	1.06	1.07	-	-
	Pack years of smoking	1.09	1.05	-	-
	Alcohol intake frequency†	1.16	1.17	1.00	0.98
WHRadjBMI	MET score	1.09	1.07	-	-
	Pack years of smoking	1.02	1.02	-	-
	Alcohol intake frequency†	1.16	1.13	1.00	0.97
Body fat percentage	MET score	1.06	1.03	-	-
	Pack years of smoking	1.04	1.03	-	-
	Alcohol intake frequency	1.09	1.09	-	-

† indicates the Lambda and LDSC intercept value more than 1.1.

MET score, Metabolic equivalent of task score; WHRadjBMI, waist to hip ratio adjusted for body mass index.

Supplementary Table 15. Two lead SNPs showing genome-wide significant G×E interactions with alcohol intake frequency in waist circumference, and body fat percentage.

Phenotype	Lifestyle factor	Chromosome	Nearest gene	SNP ID	Position[a]	Minor allele	Major allele	MAF[b] (%)	Main			G×E interaction			
									Beta	SE	P [c]	Beta	SE	P [d]	P [e]
Waist circumference	Alcohol intake frequency†	16	*FTO**	rs57292959	53799279	T	G	42.05	0.0281	0.0015	1.15×10^{-82}	0.0111	0.0015	5.13×10^{-14}	5.28×10^{-12}
Body fat percentage	Alcohol intake frequency	16	*FTO**	rs11642015	53802494	C	T	39.34	0.0558	0.0022	2.61×10^{-142}	0.0094	0.0016	4.48×10^{-9}	-

[a] Chromosomal position is based on the 1000 Genomes Project's haplotype phase 1 in NCBI build 37 (hg19).

[b] MAF is minor allele frequency calculated from subjects in this study.

[c] The *P* for the effect of association on obesity-related traits was assessed using Plink v.1.90 with adjustment for age, sex, genotyping array, and PC1~10.

[d] The *P* for the effect of interaction between genotype and lifestyle factor on obesity-related traits was assessed using Plink v.1.9.0 with adjustment for age, sex, genotyping array, and PC1~10.

e The *P* after genomic control.

* indicates the genes within which the G×E interaction SNP is located.

† indicates the GWIS after genomic control.

MAF, minor allele frequency; SE, standard error.

Supplementary Table 16. Functional annotation of two SNPs with genome-wide significant level (*P* < 4.17E-09 calculated by 5.00E-08 / 12).

Phenotype	Lifestyle factor	Chromosome	Nearest gene	SNP ID	dbSNP functional annotation	RegoulomeDB score[a]	RegoulomeDB rank[b]
Waist circumference	Alcohol intake frequency[†]	16	*FTO**	rs57292959	intronic	0.18412	7
Body fat percentage	Alcohol intake frequency	16	*FTO**	rs11642015	intronic	0.60906	4

[a] The RegulomeDB score represents a model integrating functional genomics features along with continuous values such as ChIP-seq signal, DNase-seq signal, information content change, and DeepSEA scores.

[b] RegulomeDB rank refers to the following available datatypes for a single coordinate:

4 – TF binding + DNase peak, 7 – Other.

* indicates the genes within which the G×E SNP locates

† indicates that GWIS result is applied to genomic control.

Supplementary Table 17. Previously reported association with obesity or obesity-related traits based on genetic variants within the 10 Mb flanking regions of G×E interaction SNPs.

Phenotype	Lifestyle factor	Chromosome	Nearest gene	SNP ID	BP	Reported SNP ID	Reported SNP BP	Reported traits	Reported *P* -value	Authors	PubMed ID	Title
Waist circumference	Alcohol intake frequency[†]	16	*FTO**	rs57292959	53799279	rs1421085	53800954	Body mass index	2.00E-75	Wood AR	26961502	Variants in the FTO and CDKAL1 loci have recessive effects on risk of obesity and type 2 diabetes, respectively.
Body fat percentage	Alcohol intake frequency	16	*FTO**	rs11642015	53802494	rs11642015	53802494	Body mass index	2.00E-81	Akiyama M	30239722	Genome-wide association study identifies 112 new loci for body mass index in the Japanese population.

* indicates the genes within which the G×E SNP locates

† indicates that GWIS result is applied to genomic control.

'

Supplementary Table 18. Previously reported association of two lead SNPs with obesity-related traits.

Phenotype	Lifestyle factor	Chromosome	Nearest gene	SNP ID	Reported traits	Reported *P*-value	Authors	PubMed ID	Title
Waist circumference	Alcohol intake frequency†	16	*FTO**	rs57292959	Body mass index	4.00E-07	Wood AR	26961502	Variants in the FTO and CDKAL1 loci have recessive effects on risk of obesity and type 2 diabetes, respectively.
					Heel bone mineral density	9.00E-13	Kim SK	30048462	Identification of 613 new loci associated with heel bone mineral density and a polygenic risk score for bone mineral density, osteoporosis and fracture.
					High density lipoprotein cholesterol measurement	2.00E-11	Sakaue S	34594039	A cross-population atlas of genetic associations for 220 human phenotypes.
Body fat percentage	Alcohol intake frequency	16	*FTO**	rs11642015	Smoking status measurement, systolic blood pressure	6.00E-14	Sung YJ	29455858	A Large-Scale Multi-ancestry Genome-wide Study Accounting for Smoking Behavior Identifies Multiple Significant Loci for Blood Pressure.
					Smoking status measurement, diastolic blood pressure	2.00E-10	Sung YJ	29455858	A Large-Scale Multi-ancestry Genome-wide Study Accounting for Smoking Behavior Identifies Multiple Significant Loci for Blood Pressure.
					Smoking status measurement, diastolic blood pressure	4.00E-08	Sung YJ	29455858	A Large-Scale Multi-ancestry Genome-wide Study Accounting for Smoking Behavior Identifies Multiple Significant Loci for Blood Pressure.
					Diastolic blood pressure	3.00E-06	Akiyama M	28892062	Genome-wide association study identifies 112 new loci for body mass index in the Japanese population.
					Pulse pressure measurement	3.00E-10	Takeuchi F	30487518	Interethnic analyses of blood pressure loci in populations of East Asian and European descent.
					Blood urea nitrogen measurement	4.00E-15	Takeuchi F	30487518	Interethnic analyses of blood pressure loci in populations of East Asian and European descent.

	Smoking status measurement, systolic blood pressure	1.00E-20	Takeuchi F	30487518	Interethnic analyses of blood pressure loci in populations of East Asian and European descent.
	Smoking status measurement, diastolic blood pressure	6.00E-09	Takeuchi F	30487518	Interethnic analyses of blood pressure loci in populations of East Asian and European descent.
	Smoking status measurement, systolic blood pressure	4.00E-20	Pazoki R	31409800	GWAS for urinary sodium and potassium excretion highlights pathways shared with cardiovascular traits.
	Smoking status measurement, systolic blood pressure	1.00E-13	Wang H	31453325	Genotype-by-environment interactions inferred from genetic effects on phenotypic variability in the UK Biobank.
	Grip strength measurement	8.00E-49	Wang H	31453325	Genotype-by-environment interactions inferred from genetic effects on phenotypic variability in the UK Biobank.
	Chronotype measurement	6.00E-11	Kanai M	29403010	Genetic analysis of quantitative traits in the Japanese population links cell types to complex human diseases.
	Type 2 diabetes mellitus	1.00E-35	Sung YJ	29455858	A Large-Scale Multi-ancestry Genome-wide Study Accounting for Smoking Behavior Identifies Multiple Significant Loci for Blood Pressure.
	Body mass index	9.00E-290	Sung YJ	29455858	A Large-Scale Multi-ancestry Genome-wide Study Accounting for Smoking Behavior Identifies Multiple Significant Loci for Blood Pressure.
	Age at menarche	1.00E-57	Sung YJ	29455858	A Large-Scale Multi-ancestry Genome-wide Study Accounting for Smoking Behavior Identifies Multiple Significant Loci for Blood Pressure.
	Aspartate aminotransferase measurement	7.00E-07	Sung YJ	29455858	A Large-Scale Multi-ancestry Genome-wide Study Accounting for Smoking Behavior Identifies Multiple Significant Loci for Blood Pressure.

	Serum alanine aminotransferase measurement	8.00E-20	Sung YJ	29455858	A Large-Scale Multi-ancestry Genome-wide Study Accounting for Smoking Behavior Identifies Multiple Significant Loci for Blood Pressure.
	Body fat percentage	7.00E-165	Tikkanen E	29691431	Biological Insights Into Muscular Strength
	Sex hormone-binding globulin measurement	3.00E-20	Jansen PR	30804565	Genome-wide analysis of insomnia in 1,331,010 individuals identifies new risk loci and functional pathways.
	High density lipoprotein cholesterol measurement	4.00E-16	Ishigaki K	32514122	Large-scale genome-wide association study in a Japanese population identifies novel susceptibility loci across different diseases.
	Aspartate aminotransferase measurement, serum alanine aminotransferase measurement, low density lipoprotein triglyceride measurement, body fat percentage, high density lipoprotein cholesterol measurement, sex hormone-binding globulin measurement	2.00E-157	Sakaue S	34594039	A cross-population atlas of genetic associations for 220 human phenotypes.
	Body mass index	2.00E-85	Kichaev G	30595370	Leveraging Polygenic Functional Enrichment to Improve GWAS Power.
	Blood urea nitrogen measurement	6.00E-16	Martin S	33980691	Genetic evidence for different adiposity phenotypes and their opposing influence on ectopic fat and risk of cardiometabolic disease.
	Body mass index	2.00E-81	Martin S	33980691	Genetic evidence for different adiposity phenotypes and their opposing influence on ectopic fat and risk of cardiometabolic disease.

Mean arterial pressure	1.00E-09	Martin S	33980691	Genetic evidence for different adiposity phenotypes and their opposing influence on ectopic fat and risk of cardiometabolic disease.
Systolic blood pressure	2.00E-12	Martin S	33980691	Genetic evidence for different adiposity phenotypes and their opposing influence on ectopic fat and risk of cardiometabolic disease.
Sodium measurement	7.00E-23	Martin S	33980691	Genetic evidence for different adiposity phenotypes and their opposing influence on ectopic fat and risk of cardiometabolic disease.
Body mass index	2.00E-73	Martin S	33980691	Genetic evidence for different adiposity phenotypes and their opposing influence on ectopic fat and risk of cardiometabolic disease.
Body mass index	7.00E-217	Sakaue S	34594039	A cross-population atlas of genetic associations for 220 human phenotypes.
Smoking status measurement, diastolic blood pressure	3.00E-11	Sakaue S	34594039	A cross-population atlas of genetic associations for 220 human phenotypes.
Type 2 diabetes mellitus	6.00E-27	Sakaue S	34594039	A cross-population atlas of genetic associations for 220 human phenotypes.

* indicates the genes within which the G×E interaction SNP locates

† indicates that GWIS result is applied to genomic control.

Supplementary Table 19. The eQTL information of GxE interaction two SNPs associated with obesity-related traits.

Phenotype	Lifestyle factor	Chromosome	Position	Sentinel SNP	Tested allele	Locus	Direction	Gene	eQTL *P*-value	eQTL effect size	Tissue
Waist circumference	Alcohol intake frequency†	16	53799279	rs57292959	G	*FTO*	+	*FTO*	1.10E-09	0.15	Muscle-Skeletal
Body fat percentage	Alcohol intake frequency	16	53802494	rs11642015	C	*FTO*	+	*FTO*	6.30E-08	0.14	Muscle - Skeletal
							+	*IRX3*	1.10E-05	0.36	Pancreas

† indicates that GWIS result is applied to genomic control.

Supplementary Table 20. Comparison of interaction signals for suggestive SNPs (*P* -value < 5.00E-06 in body mass index) between body mass index and waist circumference.

							Waist circumference				Body mass index		
Lifestyle factor	Chromosome	Nearrest gene	SNP ID	Position[a]	Minor allele	Major allele	MAF[b] (%)	Beta	SE	*P* [c]	Beta	SE	*P* [c]
MET score	1	*C1orf94*	rs967833	34687652	G	T	25.89	0.01111	0.002709	4.10E-05	0.00103	0.0002197	2.77E-06
	1	*SNRPE*	rs118026305	203860988	A	T	12.22	-0.01502	0.003637	3.60E-05	-0.001378	0.0002949	2.99E-06
	8	*ARHGEF10**	rs74707125	1895213	T	G	9.45	0.0168	0.004051	3.40E-05	0.001553	0.0003285	2.26E-06
	10	*ODAD2P1**	rs7075329	27600178	C	T	33.35	-0.01203	0.002504	2.00E-06	-0.01203	0.002504	1.56E-06
	10	*MIR378C*	rs7093355	132572375	T	G	11.18	0.01486	0.00372	6.50E-05	-0.001098	0.00203	6.41E-08
	14	*DAD1**	rs28624607	23040108	A	G	14.97	-0.01234	0.0033	1.84E-04	-0.01234	0.0033	1.56E-06
	16	*FTO**	rs1421085	53800954	C	T	39.43	-0.01024	0.002398	1.97E-05	-0.0009977	0.0001944	2.87E-07
	20	*MIR646HG**	rs17728377	58852966	A	C	11.11	-0.01519	0.003741	4.90E-05	-0.001406	0.0003033	3.58E-06
Alcohol intake frequency[†]	2	*SCHLAP1**	rs11677686	181561742	T	C	36.85	-0.008307	0.001514	5.02E-07	-0.0006451	0.0001228	1.80E-06
	4	*ZFP42*	rs59266038	188770462	G	T	8.296	0.0132	0.002637	4.53E-06	0.0132	0.002637	4.93E-06
	5	*CTNND2**	rs2642712	11229591	T	C	37.51	-0.007321	0.001522	1.04E-05	-0.007321	0.001522	2.12E-06
	5	*RP11-152K4.2*	rs10044831	31162365	G	A	9.407	0.0117	0.002485	1.62E-05	0.0117	0.002485	1.73E-06
	5	*C5orf60**	rs6891180	179072417	T	C	43.53	-0.007541	0.001461	2.27E-06	-0.007541	0.001464	1.84E-06
	7	*EXOC4**	rs13240997	133137753	A	C	30.5	0.007429	0.001571	1.15E-05	0.007429	0.001571	3.12E-06
	16	*FTO**	rs11642015	53802494	C	T	39.34	0.01109	0.001473	5.33E-12	0.0008286	0.0001194	7.24E-10
Pack years of smoking	1	*RP13-279N23.2*	rs72651851	19186735	T	G	8.672	0.02494	0.005229	1.86E-06	0.00213	0.0004287	6.72E-07
	1	*RNF220**	rs2236219	45101478	C	T	38.63	0.0111	0.003046	2.66E-04	0.001194	0.0002497	1.72E-06
	1	*RP11-309H21.3*	rs71637266	191111903	T	C	16.87	0.01985	0.00398	6.16E-07	0.00168	0.0003263	2.64E-07
	2	*LOC105373611**	rs72847808	129197248	C	G	18.11	-0.0128	0.003823	8.16E-04	-0.001452	0.0003133	3.60E-06
	3	*DCBLD2**	rs2439237	98572853	T	C	47.5	0.01359	0.002953	4.17E-06	0.001111	0.000242	4.45E-06
	4	*STK32B**	rs1447287	5202206	C	G	46.94	0.01223	0.00299	4.31E-05	0.001173	0.0002451	1.71E-06
	7	*POU6F2**	rs73126493	39300905	T	C	36.58	-0.0145	0.003115	3.22E-06	-0.001187	0.0002553	3.32E-06
	9	*RP11-281A20.*	rs4837532	120964337	T	G	34.87	-0.01402	0.003118	6.88E-06	-0.00124	0.0002556	1.22E-06
	9	*FAM163B**	rs113927489	136467207	G	C	11.21	-0.01898	0.004613	3.89E-05	-0.002043	0.0003781	6.60E-08
	11	*SOX6**	rs10832651	16634967	A	C	20.62	0.01314	0.003672	3.46E-04	0.001452	0.000301	1.41E-06

	14	*NRXN3**	rs8008994	79643268	T	G	27.75	-0.01134	0.003325	6.52E-04	-0.001259	0.0002725	3.82E-06
	15	*CHRNA3**	rs4243084	78911672	C	G	33.86	-0.00976	0.003093	1.61E-03	-0.00142	0.0002536	2.15E-08
	16	*CTD-2535I10.1*	rs28495409	7893107	A	G	7.607	-0.0239	0.005552	1.68E-05	-0.002233	0.0004551	9.24E-07
	16	*COG8**	rs72795276	69366986	A	G	6.487	-0.02496	0.005975	2.95E-05	-0.002322	0.0004897	2.13E-06
	17	*ASIC2**	rs2228990	32483237	G	A	19.44	0.01777	0.003707	1.65E-06	0.001564	0.0003039	2.64E-07
	17	*INC02210-CRHR1*	rs35116560	43804186	C	T	41.61	-0.01245	0.003047	4.40E-05	-0.001171	0.0002497	2.75E-06
	17	*TIMP2*	rs4789931	76926392	T	C	32.78	-0.01194	0.003146	1.47E-04	-0.001194	0.0002578	3.65E-06

[a] Chromosomal position is based on the 1000 Genomes Project's haplotype phase 1 in NCBI build 37 (hg19).

[b] MAF is minor allele frequency calculated from subjects in this study.

[c] The *P* for the effect of interaction between genotype and lifestyle factor on waist circumference or body mass index was assessed by using Plink v.1.9.0 with adjustment for age, sex, genotyping array and PC1~10.

* indicates the genes within which the GxE SNP locates.

† indicates that GWIS result is applied to genomic control.

MAF, minor allele frequency; MET score, metabolic equivalent of task score; SE, standard error.

Supplementary Table 21. Comparison of interaction signals for suggestive SNPs (*P* -value < 5.00E-06 in body mass index) between body mass index and hip circumference.

							Hip circumference				Body mass index		
Lifestyle factor	Chromosome	Nearest gene	SNP ID	Position[a]	Minor allele	Major allele	MAF[b] (%)	Beta	SE	*P* [c]	Beta	SE	*P* [c]
MET score	1	*C1orf94*	rs967833	34687652	G	T	25.89	0.01386	0.003077	6.66E-06	0.00103	0.0002197	2.77E-06
	1	*SNRPE*	rs118026305	203860988	A	T	12.22	-0.01377	0.004131	8.54E-04	-0.001378	0.0002949	2.99E-06
	8	*ARHGEF10**	rs74707125	1895213	T	G	9.45	0.02339	0.004601	3.69E-07	0.001553	0.0003285	2.26E-06
	10	*ODAD2P1**	rs7075329	27600178	C	T	33.35	-0.01134	0.002844	6.69E-05	-0.01203	0.002504	1.56E-06
	10	*MIR378C*	rs7093355	132572375	T	G	11.18	0.0103	0.004226	1.48E-02	-0.001098	0.00203	6.41E-08
	14	*DAD1**	rs28624607	23040108	A	G	14.97	-0.0122	0.003748	1.13E-03	-0.01234	0.0033	1.56E-06
	16	*FTO**	rs1421085	53800954	C	T	39.43	-0.01054	0.002724	1.09E-04	-0.0009977	0.0001944	2.87E-07
	20	*MIR646HG**	rs17728377	58852966	A	C	11.11	-0.01183	0.004249	5.36E-03	-0.001406	0.0003033	3.58E-06
Alcohol intake frequency[†]	2	*SCHLAP1**	rs11677686	181561742	T	C	36.85	0.01156	0.003197	7.79E-04	-0.0006451	0.0001228	1.80E-06
	4	*ZFP42*	rs59266038	188770462	G	T	8.296	0.009006	0.002637	1.50E-03	0.0132	0.002637	4.93E-06
	5	*CTNND2**	rs2642712	11229591	T	C	37.51	-0.005091	0.001668	4.57E-03	-0.007321	0.001522	2.12E-06
	5	*RP11-152K4.2*	rs10044831	31162365	G	A	9.407	0.007528	0.00203	5.69E-04	0.0117	0.002485	1.73E-06
	5	*C5orf60**	rs6891180	179072417	T	C	43.53	0.005428	0.001691	2.86E-03	-0.007541	0.001464	1.84E-06
	7	*EXOC4**	rs13240997	133137753	A	C	30.5	-0.008594	0.001726	3.69E-06	0.007429	0.001571	3.12E-06
	16	*FTO**	rs11642015	53802494	C	T	39.34	-0.00802	0.001821	4.25E-05	0.0008286	0.0001194	7.24E-10
Pack years of smoking	1	*RP13-279N23.2*	rs72651851	19186735	T	G	8.672	0.02815	0.005978	2.50E-06	0.00213	0.0004287	6.72E-07
	1	*RNF220**	rs2236219	45101478	C	T	38.63	0.01441	0.003482	3.50E-05	0.001194	0.0002497	1.72E-06
	1	*RP11-309H21.3*	rs71637266	191111903	T	C	16.87	0.02127	0.004551	2.97E-06	0.00168	0.0003263	2.64E-07
	2	*LOC105373611**	rs72847808	129197248	C	G	18.11	-0.01483	0.00437	6.88E-04	-0.001452	0.0003133	3.60E-06
	3	*DCBLD2**	rs2439237	98572853	T	C	47.5	0.01261	0.003376	1.87E-04	0.001111	0.000242	4.45E-06
	4	*STK32B**	rs1447287	5202206	C	G	46.94	0.01351	0.003418	7.75E-05	0.001173	0.0002451	1.71E-06
	7	*POU6F2**	rs73126493	39300905	T	C	36.58	-0.01251	0.003561	4.45E-04	-0.001187	0.0002553	3.32E-06
	9	*RP11-281A20.*	rs4837532	120964337	T	G	34.87	-0.01677	0.003564	2.55E-06	-0.00124	0.0002556	1.22E-06
	9	*FAM163B**	rs113927489	136467207	G	C	11.21	-0.02445	0.005273	3.55E-06	-0.002043	0.0003781	6.60E-08
	11	*SOX6**	rs10832651	16634967	A	C	20.62	0.01432	0.004198	6.46E-04	0.001452	0.000301	1.41E-06

	14	*NRXN3**	rs8008994	79643268	T	G	27.75	-0.01202	0.003801	1.56E-03	-0.001259	0.0002725	3.82E-06
	15	*CHRNA3**	rs4243084	78911672	C	G	33.86	-0.01743	0.003536	8.29E-07	-0.00142	0.0002536	2.15E-08
	16	*CTD-2535I10.1*	rs28495409	7893107	A	G	7.607	-0.03084	0.006347	1.19E-06	-0.002233	0.0004551	9.24E-07
	16	*COG8**	rs72795276	69366986	A	G	6.487	-0.02574	0.006831	1.64E-04	-0.002322	0.0004897	2.13E-06
	17	*ASIC2**	rs2228990	32483237	G	A	19.44	0.02118	0.004238	5.80E-07	0.001564	0.0003039	2.64E-07
	17	*INC02210-CRHR1**	rs35116560	43804186	C	T	41.61	-0.01586	0.003483	5.27E-06	-0.001171	0.0002497	2.75E-06
	17	*TIMP2*	rs4789931	76926392	T	C	32.78	-0.01455	0.003596	5.19E-05	-0.001194	0.0002578	3.65E-06

[a] Chromosomal position is based on the 1000 Genomes Project's haplotype phase 1 in NCBI build 37 (hg19).

[b] MAF is minor allele frequency calculated from subjects in this study.

[c] The *P* for the effect of interaction between genotype and lifestyle factor on hip circumference or body mass index was assessed by using Plink v.1.9.0 with adjustment for age, sex, genotyping array and PC1~10.

* indicates the genes within which the GxE SNP locates.

† indicates that GWIS result is applied to genomic control.

MAF, minor allele frequency; MET score, metabolic equivalent of task score; SE, standard error.

Supplementary Table 22. Comparison of interaction signals for suggestive SNPs (*P* -value < 5.00E-06 in body mass index) between body mass index and WHRadjBMI.

							WHRadjBMI				Body mass index		
Lifestyle factor	Chromosome	Nearest gene	SNP ID	Position[a]	Minor allele	Major allele	MAF[b] (%)	Beta	SE	*P* [c]	Beta	SE	*P* [c]
MET score	1	*C1orf94*	rs967833	34687652	G	T	25.89	0.01301	0.003147	3.60E-05	0.00103	0.0002197	2.77E-06
	1	*SNRPE*	rs118026305	203860988	A	T	12.22	-0.01378	0.004225	1.11E-03	-0.001378	0.0002949	2.99E-06
	8	*ARHGEF10**	rs74707125	1895213	T	G	9.45	0.02213	0.004706	3.00E-06	0.001553	0.0003285	2.26E-06
	10	*ODAD2P1**	rs7075329	27600178	C	T	33.35	-0.01156	0.002908	7.00E-05	-0.01203	0.002504	1.56E-06
	10	*MIR378C*	rs7093355	132572375	T	G	11.18	0.01214	0.004322	4.97E-03	-0.001098	0.00203	6.41E-08
	14	*DAD1**	rs28624607	23040108	A	G	14.97	-0.01677	0.003833	1.20E-05	-0.01234	0.0033	1.56E-06
	16	*FTO**	rs1421085	53800954	C	T	39.43	-0.01281	0.002786	4.26E-06	-0.0009977	0.0001944	2.87E-07
	20	*MIR646HG**	rs17728377	58852966	A	C	11.11	-0.0135	0.004346	1.89E-03	-0.001406	0.0003033	3.58E-06
Alcohol intake frequency[†]	2	*SCHLAP1**	rs11677686	181561742	T	C	36.85	0.01144	0.003196	9.11E-04	-0.0006451	0.0001228	1.80E-06
	4	*ZFP42*	rs59266038	188770462	G	T	8.296	0.008618	0.002636	2.46E-03	0.0132	0.002637	4.93E-06
	5	*CTNND2**	rs2642712	11229591	T	C	37.51	-0.006173	0.001668	6.06E-04	-0.007321	0.001522	2.12E-06
	5	*RP11-152K4.2*	rs10044831	31162365	G	A	9.407	0.006309	0.002029	3.98E-03	0.0117	0.002485	1.73E-06
	5	*C5orf60**	rs6891180	179072417	T	C	43.53	0.006779	0.001689	2.01E-04	-0.007541	0.001464	1.84E-06
	7	*EXOC4**	rs13240997	133137753	A	C	30.5	-0.006542	0.001726	4.47E-04	0.007429	0.001571	3.12E-06
	16	*FTO**	rs11642015	53802494	C	T	39.34	-0.006674	0.00182	6.81E-04	0.0008286	0.0001194	7.24E-10
Pack years of smoking	1	*RP13-279N23.2*	rs72651851	19186735	T	G	8.672	0.01129	0.004443	1.10E-02	0.00213	0.0004287	6.72E-07
	1	*RNF220**	rs2236219	45101478	C	T	38.63	0.003515	0.002588	1.74E-01	0.001194	0.0002497	1.72E-06
	1	*RP11-309H21.3*	rs71637266	191111903	T	C	16.87	0.0119	0.003382	4.34E-04	0.00168	0.0003263	2.64E-07
	2	*LOC105373611**	rs72847808	129197248	C	G	18.11	-0.006742	0.003249	3.80E-02	-0.001452	0.0003133	3.60E-06
	3	*DCBLD2**	rs2439237	98572853	T	C	47.5	0.008217	0.002508	1.05E-03	0.001111	0.000242	4.45E-06
	4	*STK32B**	rs1447287	5202206	C	G	46.94	0.00608	0.002539	1.66E-02	0.001173	0.0002451	1.71E-06
	7	*POU6F2**	rs73126493	39300905	T	C	36.58	-0.009677	0.002647	2.57E-04	-0.001187	0.0002553	3.32E-06
	9	*RP11-281A20.*	rs4837532	120964337	T	G	34.87	-0.005724	0.00265	3.08E-02	-0.00124	0.0002556	1.22E-06
	9	*FAM163B**	rs113927489	136467207	G	C	11.21	-0.006658	0.003918	8.93E-02	-0.002043	0.0003781	6.60E-08
	11	*SOX6**	rs10832651	16634967	A	C	20.62	0.007375	0.003121	1.81E-02	0.001452	0.000301	1.41E-06

	14	*NRXN3**	rs8008994	79643268	T	G	27.75	-0.005221	0.002826	6.47E-02	-0.001259	0.0002725	3.82E-06
	15	*CHRNA3**	rs4243084	78911672	C	G	33.86	0.000026	0.002628	9.92E-01	-0.00142	0.0002536	2.15E-08
	16	*CTD-2535I10.1*	rs28495409	7893107	A	G	7.607	-0.00735	0.004717	1.19E-01	-0.002233	0.0004551	9.24E-07
	16	*COG8**	rs72795276	69366986	A	G	6.487	-0.01626	0.005077	1.36E-03	-0.002322	0.0004897	2.13E-06
	17	*ASIC2**	rs2228990	32483237	G	A	19.44	0.007634	0.00315	1.54E-02	0.001564	0.0003039	2.64E-07
	17	*INC02210-CRHR1*†	rs35116560	43804186	C	T	41.61	-0.00564	0.002588	2.93E-02	-0.001171	0.0002497	2.75E-06
	17	*TIMP2*	rs4789931	76926392	T	C	32.78	-0.004797	0.002673	7.27E-02	-0.001194	0.0002578	3.65E-06

[a] Chromosomal position is based on the 1000 Genomes Project's haplotype phase 1 in NCBI build 37 (hg19).

[b] MAF is minor allele frequency calculated from subjects in this study.

[c] The *P* for the effect of interaction between genotype and lifestyle factor on WHRadjBMI or body mass index was assessed by using Plink v.1.9.0 with adjustment for age, sex, genotyping array and PC1~10.

* indicates the genes within which the GxE SNP locates.

† indicates that GWIS result is applied to genomic control.

MAF, minor allele frequency; MET score, metabolic equivalent of task score; SE, standard error; WHRadjBMI, waist to hip ratio adjusted for body mass index.

Supplementary Table 23. Comparison of interaction signals for suggestive SNPs (*P* -value < 5.00E-06 in body mass index) between body mass index and body fat percentage.

							Body fat percentage				Body mass index		
Lifestyle factor	Chromosome	Nearest gene	SNP ID	Position[a]	Minor allele	Major allele	MAF[b] (%)	Beta	SE	*P* [c]	Beta	SE	*P* [c]
MET score	1	*C1orf94*	rs967833	34687652	G	T	25.89	0.006537	0.001808	3.01E-04	0.00103	0.0002197	2.77E-06
	1	*SNRPE*	rs118026305	203860988	A	T	12.22	-0.008505	0.002431	4.67E-04	-0.001378	0.0002949	2.99E-06
	8	*ARHGEF10**	rs74707125	1895213	T	G	9.45	0.01183	0.002703	1.21E-05	0.001553	0.0003285	2.26E-06
	10	*ODAD2P1**	rs7075329	27600178	C	T	33.35	-0.008536	0.001672	3.33E-07	-0.01203	0.002504	1.56E-06
	10	*MIR378C*	rs7093355	132572375	T	G	11.18	0.007593	0.002485	2.24E-03	-0.001098	0.00203	6.41E-08
	14	*DAD1**	rs28624607	23040108	A	G	14.97	-0.007406	0.002204	7.79E-04	-0.01234	0.0033	1.56E-06
	16	*FTO**	rs1421085	53800954	C	T	39.43	-0.002574	0.001602	1.08E-01	-0.0009977	0.0001944	2.87E-07
	20	*MIR646HG**	rs17728377	58852966	A	C	11.11	-0.007644	0.002498	2.22E-03	-0.001406	0.0003033	3.58E-06
Alcohol intake frequency	2	*SCHLAP1**	rs11677686	181561742	T	C	36.85	-0.00362	0.00101	3.39E-04	-0.0006451	0.0001228	1.80E-06
	4	*ZFP42*	rs59266038	188770462	G	T	8.296	0.007341	0.001759	3.00E-05	0.0132	0.002637	4.93E-06
	5	*CTNND2**	rs2642712	11229591	T	C	37.51	-0.003697	0.001015	2.71E-04	-0.007321	0.001522	2.12E-06
	5	*RP11-152K4.2*	rs10044831	31162365	G	A	9.407	0.007993	0.001658	1.44E-06	0.0117	0.002485	1.73E-06
	5	*C5orf60**	rs6891180	179072417	T	C	43.53	-0.0038	0.000975	9.76E-05	-0.007541	0.001464	1.84E-06
	7	*EXOC4**	rs13240997	133137753	A	C	30.5	0.004089	0.001048	9.56E-05	0.007429	0.001571	3.12E-06
	16	*FTO**	rs11642015	53802494	C	T	39.34	0.009405	0.001604	4.48E-09	0.0008286	0.0001194	7.24E-10
Pack years of smoking	1	*RP13-279N23.2*	rs72651851	19186735	T	G	8.672	0.01485	0.003491	2.10E-05	0.00213	0.0004287	6.72E-07
	1	*RNF220**	rs2236219	45101478	C	T	38.63	0.009312	0.002034	5.00E-06	0.001194	0.0002497	1.72E-06
	1	*RP11-309H21.3*	rs71637266	191111903	T	C	16.87	0.009572	0.002658	3.17E-04	0.00168	0.0003263	2.64E-07
	2	*LOC105373611**	rs72847808	129197248	C	G	18.11	-0.007736	0.002553	2.45E-03	-0.001452	0.0003133	3.60E-06
	3	*DCBLD2**	rs2439237	98572853	T	C	47.5	0.00627	0.001972	1.48E-03	0.001111	0.000242	4.45E-06
	4	*STK32B**	rs1447287	5202206	C	G	46.94	0.008038	0.001996	5.70E-05	0.001173	0.0002451	1.71E-06
	7	*POU6F2**	rs73126493	39300905	T	C	36.58	-0.00724	0.002081	5.03E-04	-0.001187	0.0002553	3.32E-06
	9	*RP11-281A20.*	rs4837532	120964337	T	G	34.87	-0.009241	0.002083	9.00E-06	-0.00124	0.0002556	1.22E-06
	9	*FAM163B**	rs113927489	136467207	G	C	11.21	-0.0115	0.003079	1.88E-04	-0.002043	0.0003781	6.60E-08
	11	*SOX6**	rs10832651	16634967	A	C	20.62	0.01131	0.002453	4.00E-06	0.001452	0.000301	1.41E-06

	14	*NRXN3**	rs8008994	79643268	T	G	27.75	-0.007612	0.002221	6.09E-04	-0.001259	0.0002725	3.82E-06
	15	*CHRNA3**	rs4243084	78911672	C	G	33.86	-0.007275	0.002065	4.27E-04	-0.00142	0.0002536	2.15E-08
	16	*CTD-2535I10.1*	rs28495409	7893107	A	G	7.607	-0.01676	0.003705	6.00E-06	-0.002233	0.0004551	9.24E-07
	16	*COG8**	rs72795276	69366986	A	G	6.487	-0.0165	0.003991	3.60E-05	-0.002322	0.0004897	2.13E-06
	17	*ASIC2**	rs2228990	32483237	G	A	19.44	0.009363	0.002475	1.55E-04	0.001564	0.0003039	2.64E-07
	17	*INC02210-CRHR1**	rs35116560	43804186	C	T	41.61	-0.008259	0.002034	4.90E-05	-0.001171	0.0002497	2.75E-06
	17	*TIMP2*	rs4789931	76926392	T	C	32.78	-0.007836	0.002101	1.92E-04	-0.001194	0.0002578	3.65E-06

[a] Chromosomal position is based on the 1000 Genomes Project's haplotype phase 1 in NCBI build 37 (hg19).

[b] MAF is minor allele frequency calculated from subjects in this study.

[c] The *P* for the effect of interaction between genotype and lifestyle factor on body fat percentage or body mass index was assessed by using Plink v.1.9.0 with adjustment for age, sex, genotyping array and PC1~10.

* indicates the genes within which the GxE SNP locates.

† indicates that GWIS result is applied to genomic control.

MAF, minor allele frequency; MET score, metabolic equivalent of task; SE, standard error; WHRadjBMI, waist to hip ratio adjusted for body mass index.
